# Synthesis and antitumor activity of mono and digold(i) alkynyl complexes with oligo(ethylene glycol)methylether

**DOI:** 10.1039/d5ra08951d

**Published:** 2026-02-03

**Authors:** Yanyan Zeng, Fei Zheng, Lingyu Jin, Qiu Mei Chen, Ping Zhou, Xiaoqing Mou, Xiang Hua Wu, Jun Feng Zhang, Wen Xiu Ren

**Affiliations:** a College of Chemistry and Chemical Engineering, Yunnan Normal University Kunming 650500 China chxhwu@sina.com junfengzhang78@126.com; b Department of Radiology, The Affiliated Hospital, Southwest Medical University NO.25, Taiping Road, Jiangyang District Luzhou 646000 Sichuan China xrenwenxiux@swmu.edu.cn; c The Affiliated Dazu's Hospital of Chongqing Medical University Chongqing 402360 China; d Precision Imaging and Intelligent Analysis Key Laboratory of Luzhou, Southwest Medical University Luzhou 646000 Sichuan China

## Abstract

Preparation and characterization of a series of oligo(ethylene glycol)methylether functionalized alkynyl gold(i) complexes capped with AuPPh_3_ (1a–1d) or dppfAu_2_ (dppf, 1,1′-*bis*(diphenyphosphino)ferrocene) (2a–2d) have been accomplished. The structures of 1b and 1c were established by X-ray crystallography. Their *in vitro* antitumor activities were measured by the CCK8 method against A549 and HeLa cells. The studies indicated that the cytotoxic activity *in vitro* was fine-tuned by modification of both the gold(i) centers and the oligo(ethylene glycol)methylether ancillary ligands. Compared to the dppfAu_2_ series, the AuPPh_3_ series showed better cytotoxicity. Especially, complex 4-(OCH_2_CH_2_OCH_2_CH_2_OCH_3_)C_6_H_4_C≡CAuPPh_3_ (1d) displayed strong anticancer activity toward both cancer cells due to the strong inhibition of thioredoxin reductase (TrxR).

## Abstract 2

In this paper, novel oligo(ethylene glycol)methylether functionalized alkynyl gold(i) complexes capped with AuPPh_3_ or dppfAu_2_ were synthesized. The AuPPh_3_ series showed better cytotoxicity and were powerful inhibitors of TrxR.

## Graphical abstract

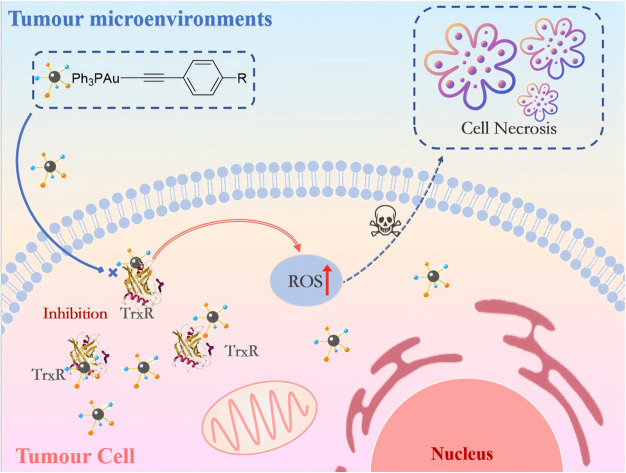

## 1. Introduction

Among non-platinum metal-complex based anti-tumor drugs, gold complexes have received increased attention in recent years.^1–4^ Due to the suitable stability and strong antiproliferative potency, alkynyl gold(i) complexes are becoming a rising star in the field of developing gold-based antitumor agents.^5^ The activity of alkynyl gold(i) complexes can be fine-tuned by substituting ligands in the gold centre, thereby enhancing the affinity of gold(i) toward sulfur and selenium moities in biologically important substrates.

Thioredoxin reductase (TrxR) is a major REDOX regulator in mammalian cells, often overexpressed in many cancer cells, and the critical target enzyme for the anticancer effects of alkynyl gold(i) complexes.^6^ Ott's group developed a series of alkynyl gold(i) complexes with reinforced anticancer efficiency. Among them, alkynyl (triphenylphosphine) gold(i) complexes incorporating either anisole or benzyl ether units could act strongly against TrxR.^7^ Xanthine-derived alkynyl (triphenylphosphine) gold(i) complexes displayed multiple functions, including selective cytotoxicity, antimetastatic, and antiangiogenic properties.^8^ Furthermore, alkynylgold(i)(NHC) complexes with methoxy substitution enhanced dipole moment correlation possessed significant antiproliferative effects against different cell lines.^9^ Our previous work also showed that the antitumor activities of digold-alkynyl complexes could be increased by introducing 1,4-diethenylbenzene bridge containing two oligo(ethylene glycol)methylether side chains.^10^ In addition, ferrocene ligands are good candidates for constructing heterometallic complexes due to the low toxicity, high lipophilicity, and unique electrochemical behavior.^11^ When combined with Pt(ii),^12^ Ru(ii),^13^ and Cu(i),^14^ the ferrocene-based heterometallic complexes obtained have better biocompatibility and anti-proliferation properties.

Inspired by this, in this paper, eight oligo(ethylene glycol)methylether functionalized alkynyl gold(i) complexes capped with AuPPh_3_ or dppfAu_2_ (dppf, 1,1′-bis(diphenyphosphino)ferrocene) have been synthesized. *In vitro* antitumor activities of these alkynyl (phosphine) gold(i) complexes against A549 and HeLa cells were investigated. Moreover, the inhibition of TrxR was studied in order to gain preliminary mechanistic insight.

## 2. Results and discussion

### 2.1 Chemistry

**Scheme 1:**
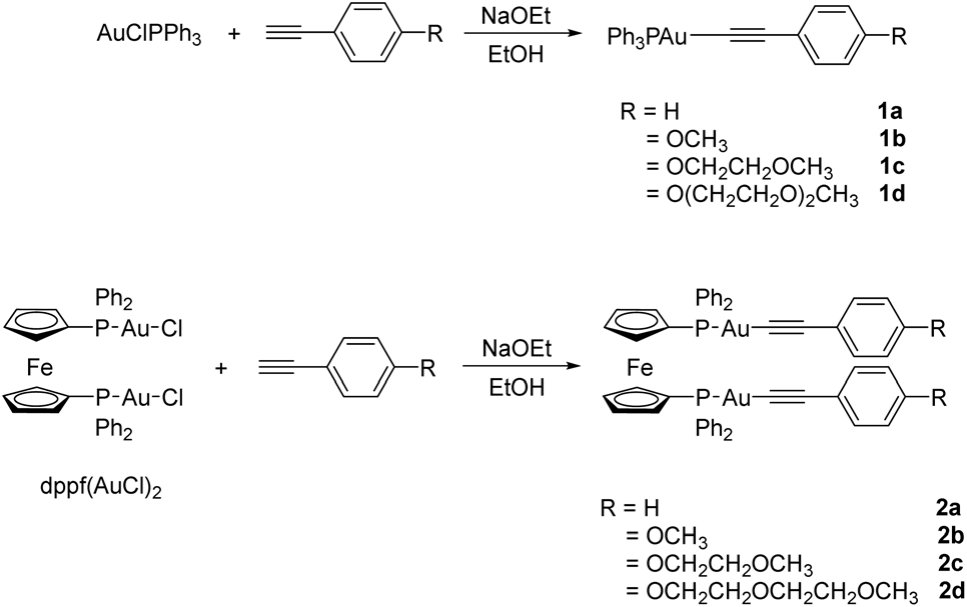
Synthesis of mono and digold(i) alkynyl complexes 1a–d and 2a–d.

The structures and general synthetic routes of mono and digold(i) alkynyl complexes are outlined in Scheme 1. The alkynyl(phosphane)gold(i) complexes were obtained by the substituted alkynyl derivatives reacting with the gold complexes AuCl(PPh_3_) and dppf(AuCl)_2_, respectively, from moderate to good yield in the presence of NaOEt. These complexes were characterized by NMR and MS, which are given in the SI (Fig. S1–S32). In addition, the crystal structures of 1b and 1c were determined by X-ray diffraction analysis.

### 2.2 X-ray diffraction studies

**Fig. 1:**
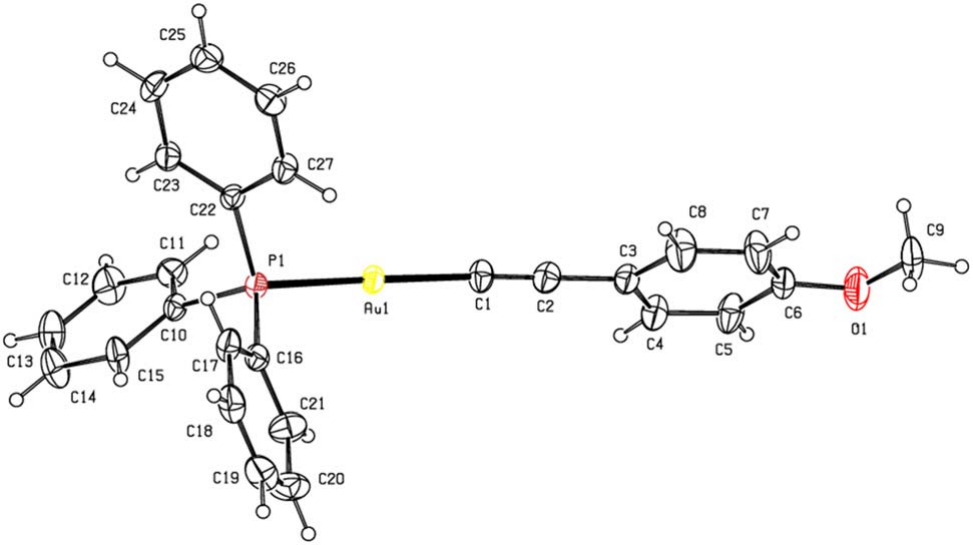
Molecular structure of 1b with displacement ellipsoids drawn at the 30% probability level.

**Fig. 2:**
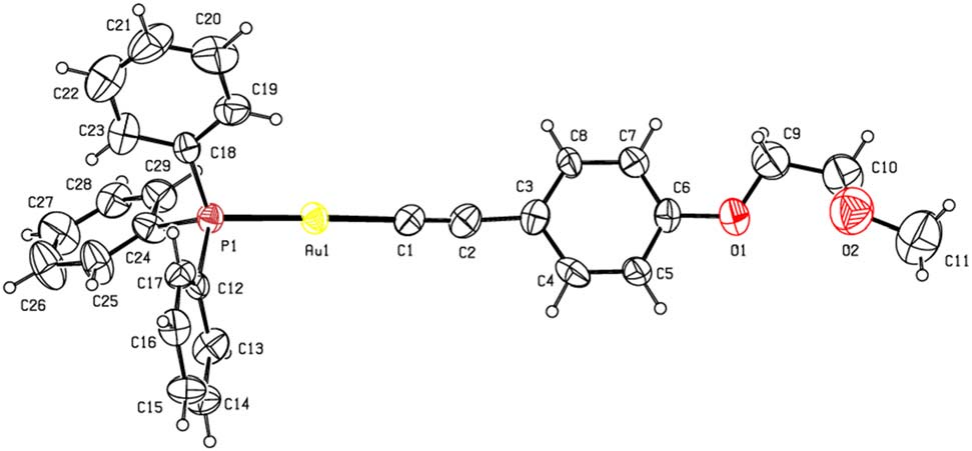
Molecular structure of 1c with displacement ellipsoids drawn at the 30% probability level.

The single-crystal X-ray structures of 1b and 1c are depicted in Fig. 1 and 2, respectively. Full crystallographic data and selected bond metrics are provided in Tables S1 and S2 of the SI. In the structures of 1b and 1c, the ≡C–Au–P angles of 173.22(18)° (1b) and 175.1(3)° (1c) show that the Au centers are approximately linearly coordinated.^10,21–24^ The C≡C bond lengths are 1.191(9) Å (1b) and 1.16(2) Å (1c), which are typical of terminal alkynyl gold(i) complexes.^10,15–18^ The Au–P and Au–C bond lengths in 1b and 1c are comparable to those found in known alkynyl(phosphane)gold(i) complexes.^10,15–18^

### 2.3 Cytotoxic activity *in vitro* and inhibition of TrxR

The *in vitro* cytotoxicity of mono- and digold(i) complexes was assessed against A549 (human lung carcinoma) and HeLa (human cervical carcinoma) cell lines using the CCK-8 assay, with cisplatin (CDDP) serving as the positive control; IC_50_ values are summarized in Table 1. A549 (human lung carcinoma) and HeLa (human cervical carcinoma) cell lines” were purchased from Shanghai Mingjing Biotechnology Co., Ltd.

**Table 1:** *In vitro* antiproliferative activity of mono and digold(i) complexes against two cancer cell lines

Compound	Antiproliferative activity (IC_50_^a^, µM)	
	A549	HeLa
AuCl-PPh_3_	5.07 ± 0.11	2.56 ± 0.49
1a	9.68 ± 0.03	2.52 ± 0.27
1b	>100	>100
1c	13.13 ± 0.16	3.18 ± 0.05
1d	8.82 ± 0.06	2.28 ± 0.02
dppf(AuCl)_2_	9.21 ± 0.11	7.76 ± 0.26
2a	>100	>100
2b	>100	>100
2c	>100	57.65 ± 7.65
2d	39.51 ± 1.06	29.33 ± 0.98
CDDP	20.26 ± 1.20	21.25 ± 1.15

a Antiproliferative activity was evaluated after 24 h exposure of the cell lines to the complexes and is expressed as the IC_50_ value. Data are reported as the mean ± SD from three independent dose-response curves.

As shown in Table 1 and Fig. S33, the antitumor activity of mono gold(i) complexes except 1b are highly cytotoxic toward A549 and HeLa cells. The antiproliferative activity of complexes 1a, 1c and 1d against A549 cells is superior to that of the positive control drug cisplatin, in which the IC_50_ of complex 1d is about 2.3 times higher than that of cisplatin. However, complexes 1a, 1c and 1d were less effective in A549 cells in comparison to AuClPPh_3_. For HeLa cell lines, complexes 1a, 1c and 1d exhibited promising antiproliferative activity with the highest activity shown by complex 1d (IC_50_ 2.28 ± 0.02 µM), in which the level of activity was about 9.3 times higher than that of cisplatin. Moreover, complexes 1a and 1d were more effective in HeLa cells in comparison to AuClPPh_3_. The IC_50_ values of complexes 1a, 1c and 1d were further reduced after 48 h of incubation (Fig. S34). Compared to the mono gold(i) series, when the oligo(ethylene glycol)methylether moiety was introduced into alkynyl dppf digold(i) complexes, the antitumor activities of the resulted complexes 2a–2d were reduced significantly. The observed result can probably be attributed to the smaller size of the triphenylphosphane ligand in 1, which facilitates cellular uptake of the triphenylphosphane gold complexes, resulting in higher activity.^8^ The alkynyl (phosphine) gold(i) complexes exhibited superior activity to the previously reported *N*-heterocyclic carbene alkynylgold(i) complexes.^19,20^ Compared with the corresponding alkynylgold(i) complexes, phosphine ligands appear to be more effective. Notably, the increase in the length of oligo(ethylene glycol)methylether positively influenced the cell activities in the selected cell lines. This is basically consistent with the results reported in the relevant literature.^10,21^

As stated above, TrxR may be one of critical pharmacological target for alkynyl gold(i) species. On the basis of the cytotoxicity, complexes 1a, 1c, and 1d were selected as examples to study the inhibition of TrxR. As shown in Table 2 and Fig. S35, complex 1d displayed the strongest inhibition of TrxR, with an EC_50_ value in the submicromolar range (0.77 µM), which is lower than that of auranofin and the alkynyl gold(i) species reported by Ott.^7,22^ These results indicated TrxR can be considered to be one of the main cellular targets for oligo(ethylene glycol)methylether functionalized mono alkynyl triphenylphosphine gold(i) complexes.

**Table 2:** Inhibition of TrxR

Complex	EC_50_ TrxR^a^ (uM)
1a	33.12
1c	50.49
1d	0.77

a The EC_50_ values were calculated as the concentration of compound decreasing the enzymatic activity of the untreated control by 50%.

Overall, these studies suggested that the oligo(ethylene glycol)methylether functionalized alkynyl (phosphine) gold(i) complexes showed excellent to good antiproliferative activity and strong inhibition of TrxR, which may provide a promising lead for the development of antitumor metallodrug.

## 3. Conclusions

In summary, a series of mono and digold(i) alkynyl complexes functionalized with oligo(ethylene glycol)methylether have been synthesized and characterized. Their antitumor activity *in vitro* have been investigated against two cancers cell lines. It is found that the cytotoxic activity *in vitro* was decided by the gold(i) centers and the oligo(ethylene glycol)methylether ancillary ligands. The present work demonstrates that the oligo(ethylene glycol)methylether functionalized alkynyl (triphenylphosphine) gold(i) complex 1d was identified as the most promising candidate due to its high cytotoxicity and strong inhibition of TrxR. Beyond our expectation, alkynyl dppf digold(i) complexes did not exhibit good anticancer performance. The relevant reasons remain to be further explored. This study was initiated in the frame of ongoing studies aimed at developing new organometallic gold(i) complexes as potential anticancer metallodrug.

## 4. Experimental section

### 4.1 Chemistry

#### 4.1.1 General materials

All manipulations were performed at room temperature under a nitrogen atmosphere using standard Schlenk techniques unless otherwise noted. ^1^H, ^13^C, and ^31^P NMR spectra were recorded on a Varian Mercury Plus 500 spectrometer (500 MHz). ^1^H and ^13^C chemical shifts are referenced TMS, and ^31^P chemical shifts are referenced to 85% H_3_PO_4_. Mass spectra were recorded with a Bruker (micrOTOF II). Solvents were pre-dried, distilled and degassed prior to use. The reagents ethynyltrimethylsilane, phenylacetylene, and 4-Methoxyphenylacetylene were purchased from Alfa Aesar. Others were commercially available. The starting materials AuCl(PPh_3_),^23^ Dppf(AuCl)_2_,^24^ 1-ethynyl-4-(2-methoxyethoxy)benzene,^25^ and 1-ethynyl-4-(2-(2-methoxyethoxy)ethoxy)benzene^25^ were prepared by the procedures described in literature methods.

#### 4.1.2 General synthesis of mono gold(i) complexes 1a–1d

A ethanol solution (20 mL) of phenylacetylene derivatives (0.3 mmol) and an excess of Na (23 mg, 1.0 mmol) was stirred for 30 min. Au(PPh_3_)Cl (0.3 mmol) was then added, and the mixture was stirred for 3 h in the dark. The solid was collected by filtration.

#### 4.1.3 (PPh_3_)Au–C≡C–C_6_H_5_ (1a)

White solid, yield: 106 mg, 61%. ^1^H NMR (500 MHz, CDCl_3_): *δ* 7.54 (d, *J* = 32.7 Hz, 20H). ^13^C NMR (125 MHz, CDCl_3_) *δ* 134.38, 134.27, 132.38, 132.14, 132.06, 131.57, 129.97, 129.52, 129.19, 129.10, 128.54, 128.45, 127.94, 126.79. ^31^P NMR (200 MHz, CDCl_3_): *δ* 42.19. TOF-MS, *m*/*z*: calcd, 560.0968; found, 583.0911 ([M + Na^+^]).

#### 4.1.4 (PPh_3_)Au–C≡C–C_6_H_4_–OCH_3_-*p* (1b)

White solid, yield: 96 mg, 54%. ^1^H NMR (500 MHz, CDCl_3_) *δ* 7.61–7.45 (m, 17H), 6.82 (d, *J* = 8.5 Hz, 2H), 3.81 (s, 3H). ^13^C NMR (125 MHz, CDCl_3_) *δ* 158.54, 134.39, 134.28, 133.67, 131.52, 131.51, 130.07, 129.63, 129.16, 129.07, 113.57, 55.18. ^31^P NMR (200 MHz, CDCl_3_) *δ* 42.36. TOF-MS, *m*/*z*: calcd, 590.1074, found, 591.1144 ([M + H^+^]).

#### 4.1.5 (PPh_3_)Au–C≡C–C_6_H_4_–OCH_2_CH_2_OCH_3_-*p* (1c)

White solid, yield: 109 mg, 58%.^1^H NMR (500 MHz, CDCl_3_) *δ* 7.59–7.46 (m, 17H), 6.86–6.81 (m, 2H), 4.12 (dd, *J* = 5.4, 4.0 Hz, 2H), 3.76 (dd, *J* = 5.4, 4.0 Hz, 2H), 3.47 (d, *J* = 5.4 Hz, 3H). ^13^C NMR (125 MHz, DMSO) *δ* 157.75, 134.39, 134.28, 133.09, 132.33, 130.10, 130.01, 114.94, 70.79, 67.35, 58.61. ^31^P NMR (200 MHz, CDCl_3_): *δ* 42.32. TOF-MS, *m*/*z*: calcd, 634.1336, found, 635.1409 ([M + H^+^]).

#### 4.1.6 (PPh_3_)Au–C≡C–C_6_H_4_–O(CH_2_CH_2_O)_2_CH_3_-*p* (1d)

White solid, yield: 117 mg, 58%.^1^H NMR (500 MHz, CDCl_3_) *δ* 7.63–7.41 (m, 17H), 6.82 (d, *J* = 8.8 Hz, 2H), 4.16–4.13 (m, 2H), 3.88–3.86 (m, 2H), 3.74–3.72 (m, 2H), 3.60–3.58 (m, 2H), 3.41 (d, *J* = 2.8 Hz, 3H). ^13^C NMR (125 MHz, CDCl_3_) *δ* 134.38, 134.27, 133.65, 131.54, 131.52, 130.02, 129.57, 129.17, 129.08, 114.28, 71.96, 70.74, 69.73, 67.36, 59.07, 41.01. ^31^P NMR (200 MHz, CDCl_3_) *δ* 42.32. TOF-MS, *m*/*z*: calcd, 678.1598, found, 679.1676 ([M + H^+^]).

#### 4.1.7 General synthesis of digold(i) complexes 2a–2d

A ethanol solution (20 mL) of phenylacetylene derivatives (0.6 mmol) and an excess of Na (23 mg, 1.0 mmol) was stirred for 30 min. dppf(AuCl)_2_ (0.3 mmol) was then added, and the mixture was stirred for 3 h in the dark. The solid was collected by filtration and washed with diethyl ether.

2a: Pale yellow solid, yield: 235 mg, 61%. ^1^H NMR (500 MHz, CDCl_3_) *δ* 7.60–7.39 (m, 26H), 7.25 (dd, *J* = 15.1, 7.9 Hz, 4H), 4.77 (s, 4H), 4.32 (s, 4H). ^13^C NMR (125 MHz, CDCl_3_) *δ* 133.62, 133.51, 132.34, 131.73, 129.11, 129.02, 127.97, 126.84. ^31^P NMR (200 MHz, CDCl_3_) *δ* 36.85. TOF-MS, *m*/*z*: calcd, 1150.1129, found,1167.1385 ([M + NH_4_^+^]).

2b: Pale yellow solid, yield: 230 mg, 68%. ^1^H NMR (500 MHz, CDCl_3_) *δ* 7.58–7.41 (m, 24H), 6.82 (d, *J* = 8.6 Hz, 4H), 4.76 (s, 4H), 4.31 (s, 4H), 3.82 (s, 6H). ^13^C NMR (125 MHz, CDCl_3_) *δ* 158.58, 133.66, 131.43, 129.01, 128.92, 117.10, 113.61, 75.08, 74.77, 55.20. ^31^P NMR (200 MHz, CDCl_3_) *δ* 36.85. TOF-MS, *m*/*z*: calcd, 1210.1340, found, 1210.134 ([M^+^]).

2c: Pale yellow solid, yield: 225 mg, 65%. ^1^H NMR (500 MHz, CDCl_3_) *δ* 7.56–7.42 (m, 24H), 6.92–6.86 (m, 4H), 4.75 (d, *J* = 1.0 Hz, 4H), 4.30 (dt, *J* = 3.5, 1.9 Hz, 4H), 4.15 (dd, *J* = 5.4, 3.9 Hz, 4H), 3.78 (dd, *J* = 5.4, 3.9 Hz, 4H), 3.48 (s, 6H). ^13^C NMR (125 MHz, CDCl_3_) *δ* 157.77, 133.72, 133.63, 131.44, 129.01, 128.92, 117.34, 114.28, 75.14, 75.07, 74.87, 74.77, 71.00, 67.20, 59.20. ^31^P NMR (200 MHz, CDCl_3_) *δ* 36.85.TOF-MS, *m*/*z*: calcd, 1298.1865, found, 1321.1754 ([M + Na^+^]).

2d: Pale yellow solid, yield: 253 mg, 70%. ^1^H NMR (500 MHz, CDCl_3_) *δ* 7.58–7.40 (m, 24H), 6.85 (dd, *J* = 22.4, 8.7 Hz, 4H), 4.75 (s, 4H), 4.30 (s, 4H), 4.19–4.12 (m, 4H), 3.88 (dd, *J* = 9.9, 5.1 Hz, 4H), 3.77–3.70 (m, 4H), 3.60 (dt, *J* = 6.3, 3.2 Hz, 4H), 3.41 (s, 6H). ^13^C NMR (125 MHz, CDCl_3_) *δ* 157.77, 133.73, 133.62, 131.43, 129.01, 128.92, 117.29, 114.30, 75.14, 75.09, 74.87, 74.77, 71.95, 70.74, 69.74, 67.37, 59.07. ^31^P NMR (200 MHz, CDCl_3_) *δ* 36.81. TOF-MS, *m*/*z*: calcd, 1386,2389, found, 1409.2288 ([M + Na^+^]).

### 4.2 Crystallographic analysis

Crystals suitable for X-ray diffraction were grown from a dichloromethane of solution 1b and 1c layered with hexane. Diffraction data were collected on a Bruker P4 diffractometer with Mo Kα radiation (*λ* = 0.71073 Å). The structure was solved by direct methods (SHELXS-97) and refined by full-matrix least-squares on F^2^ using SHELXL-97 within the WINGX suite.^26,27^ Crystal data and refinement details are summarized in Table S1; selected bond lengths and angles are listed in Table S2.

### 4.3 Cell viability assay

Cell viability of the complex against A549 and HeLa cells was assessed as follows. A549 cells were maintained in Ham's F-12K and HeLa cells in DMEM, each supplemented with 10% fetal bovine serum and 1% penicillin–streptomycin at 37 °C under 5% CO_2_/95% air. Cells were seeded at 6000 cells per well in 96-well plates and allowed to adhere for 24 h. After medium removal, fresh medium containing serial dilutions of the complex was added and incubation continued for 24 h. CCK-8 reagent (10 µL) was then added to each well and the plates were incubated for 30–60 min. Absorbance at 450 nm was measured on a Multiskan Sky microplate reader (USA). Viability was calculated as (mean absorbance of treated wells/mean absorbance of control wells) × 100%. Data are presented as mean ± SD of three independent experiments.

### 4.4 TrxR inhibition

The inhibition of TrxR was determined by described procedures with some minor modifications.^28^ Commercially available rat liver TrxR (from Sigma-Aldrich) was used and diluted with distilled water to achieve 2.0 U mL^−1^. The complexes were freshly dissolved as stock solutions in DMSO. To each, 20 µL aliquots of the enzyme solution, each 20 µL of potassium phosphate buffer, pH 7.0, containing the compounds in graded concentrations (0.15625, 0.3125, 0.625, 1.25, 2.5, 5, 10, 20, 40, 80, 100 µmol L^−1^) or DMSO (control), were added, and the resulting solutions (final concentration of DMSO, 0.5% v/v) were incubated with moderate shaking for 75 min at 37 °C in a 96-well plate. To each well, an amount of 140 µL of the reaction mixture was added, and the reaction was started by addition of 20 µL of a 20 mM ethanolic DTNB solution. After proper mixing, the formation of TNB was monitored with a microplate reader (MULTISKAN Sky, USA) at 412 nm at 30 s intervals for 30 min. For each tested compound the non interference with the assay components was confirmed by a negative control experiment using an enzyme free solution.

## Author contributions

Conceptualization: X. H. Wu, W. X. Ren; data curation: Y. Zeng, F. Zheng, Q. M. Chen; formal analysis: L. Jin, X. H. Wu; funding acquisition: J. F. Zhang, W. X. Ren; investigation: P. Zhou; methodology: X. H. Wu, W. X. Ren; project administration: J. F. Zhang, W. X. Ren; software: X. Mou; supervision: J. F. Zhang, W. X. Ren; writing – original draft: X. H. Wu; writing – review and editing: X. H. Wu, W. X. Ren.

## Conflicts of interest

There are no conflicts to declare.

## Supplementary Material

RA-016-D5RA08951D-s001

RA-016-D5RA08951D-s002

## Data Availability

CCDC 2504135 (1b) and 2504116 (1c) contain the supplementary crystallographic data for this paper.^29*a*,*b*^ The authors confirm that the data supporting the findings of this study are available within the article and its supplementary information (SI) or from the corresponding author upon request. Supplementary information: crystallographic details, NMR spectra, MS spectra, antiproliferative activity, and inhibition of TrxR. See DOI: https://doi.org/10.1039/d5ra08951d.
