# Supplementary material for: Synthesis and antitumor activity of mono and digold(i) alkynyl complexes with oligo(ethylene glycol)methylether
Source: RSC Adv. 2026 Feb 3;16(8):7092–6. doi: 10.1039/d5ra08951d (PMC12865784; doi:10.1039/d5ra08951d)
Supplement: RA-016-D5RA08951D-s001 [file RA-016-D5RA08951D-s001.pdf]

Supplementary Information (SI) for RSC Advances.
This journal is © The Royal Society of Chemistry 2026

*Supplementary data*

# Synthesis and antitumor activity of mono and digold(I) alkynyl complexes with oligo(ethylene glycol)methylether

Yanyan Zeng[b,c,#], Fei Zheng[a,#] Lingyu Jin[b,#], Qiu Mei Chen[a], Ping Zhou[b], Xiaoqing Mou[b], Xiang Hua Wu[a,*], Jun Feng Zhang[a,*], and Wen Xiu Ren[b,d*]

[a]Yunnan Key Laboratory of Modern Separation Analysis and Substance Transformation, College of Chemistry and Chemical Engineering, Yunnan Normal University, Kunming 650500, China

[b]Department of Radiology, the Affiliated Hospital of Southwest Medical University, Luzhou 646000, China

[c]The Affiliated Dazu's Hospital of Chongqing Medical University,Chongqing 402360, China

[d]Precision Imaging and Intelligent Analysis Key Laboratory of Luzhou, Southwest Medical University, Luzhou, 646000, Sichuan, China

E-mail addresses: chxhwu@sina.com (X. H. Wu), junfengzhang@126.com (J. F. Zhang), xrenwenxiux@swmu.edu.cn (W. X. Ren)

Y. Zeng, F. Zheng and L. Jin contributed equally to this work.

**Supplemental Materials**

**1. Crystallographic details**

**2. NMR and MS spectra**

**3. Antiproliferative activity**

**4. Inhibition of TrxR**

## 1. Crystallographic details

**Table S1**. Crystal data and structure refinement for complexes **1b** and **1c**

| Complex | **1b** | **1c** |
|---|---|---|
| Formula | $C_{54}H_{44}Au_2O_2P_2$ | $C_{58}H_{52}Au_2O_4P_2$ |
| Fw | 1180.76 | 1268.87 |
| Temp(K) | 293(2) | 293(2) |
| Wavelength(Å) | 0.71073 | 0.71073 |
| Crystal system | Triclinic | Triclinic |
| Space group | P1 | P1 |
| *a*(Å) | 10.033 | 10.434(2) |
| *b*(Å) | 10.151 | 10.785(2) |
| *c*(Å) | 13.402 | 13.251(3) |
| $\alpha$(°) | 102.15 | 92.47(3) |
| $\beta$(°) | 106.83 | 110.71(3) |
| $\gamma$(°) | 111.15 | 110.25(3) |
| $V$(Å$^3$) | 1140.0 | 1285.2(6) |
| $Z$ | 1 | 1 |
| Density (calculated)(Mg/m$^3$) | 1.720 | 1.639 |
| Absorption coefficient(mm$^{-1}$) | 6.538 | 5.809 |
| F(000) | 572 | 620 |
| $\theta$ Range(°) | 3.061-24.999 | 6.394-49.994 |
| | -11<=h<=11 | -12 <=h<=12 |
| Index ranges | -11<=k<=12 | -11<=k<=12 |
| | -15<=l<=15 | -15<=l<=15 |
| Reflections collected | 8962 | 9554 |
| Independent reflec. | 3987 [R(int) = 0.0694] | 4431 [R(int) = 0.0936] |
| Data / restraints / parameters | 3987 / 0 / 272 | |
| Goodness-of-fit on F$^2$ | 1.028 | 1.036 |
| Final *R* indices [I>2sigma(I)] | $R_1$ = 0.0456, | $R_1$ = 0.0709, $wR_2$ = |

| | | |
|---|---|---|
| | $wR_2 = 0.1069$ | 0.1812 |
| $R$ indices (all data) | $R_1 = 0.0474$, $wR_2 = 0.1082$ | $R_1 = 0.0992$, $wR_2 = 0.2190$ |
| Largest diff. peak and hole | 2.56 and -2.30 e. Å$^{-3}$ | 1.28 and -2.07 e. Å$^{-3}$ |

**Table S2.** Selected bond lengths (Å) and angles (deg) for complexes **1b** and **1c**

| complex **1b** | | complex **1c** | |
|---|---|---|---|
| Au(1)-P(1) | 2.2821(15) | Au(1)-P(1) | 2.274(4) |
| Au(1)-C(1) | 2.012(7) | Au(1)-C(1) | 2.035(17) |
| C(1)-C(2) | 1.191(9) | C(1)-C(2) | 1.16(2) |
| C(9)-O(1) | 1.408(11) | C(6)-O(1) | 1.386(18) |
| C(1)-Au(1)-P(1) | 173.22(18) | C(1)-Au(1)-P(1) | 175.1(3) |
| C(2)-C(1)-Au(1) | 174.1(6) | C(2)-C(1)-Au(1) | 169.0(12) |

## 2. NMR and MS spectra

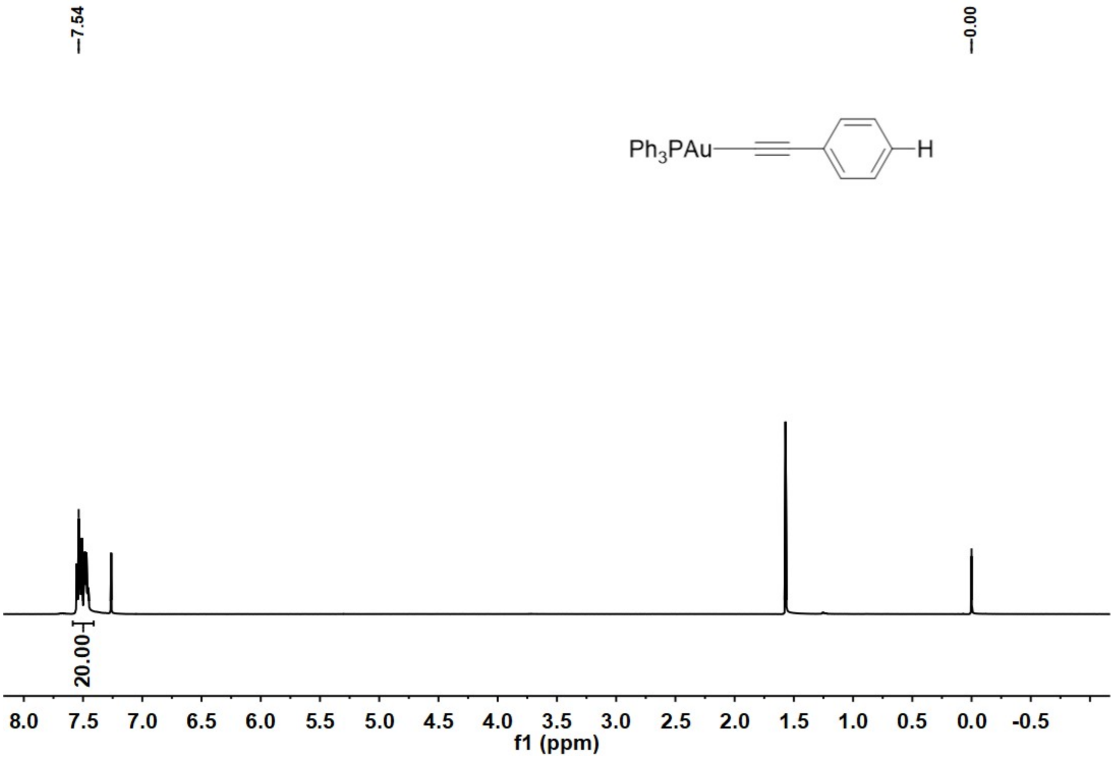


**Fig. S1** $^1$H NMR spectra of complex **1a** in $CDCl_3$

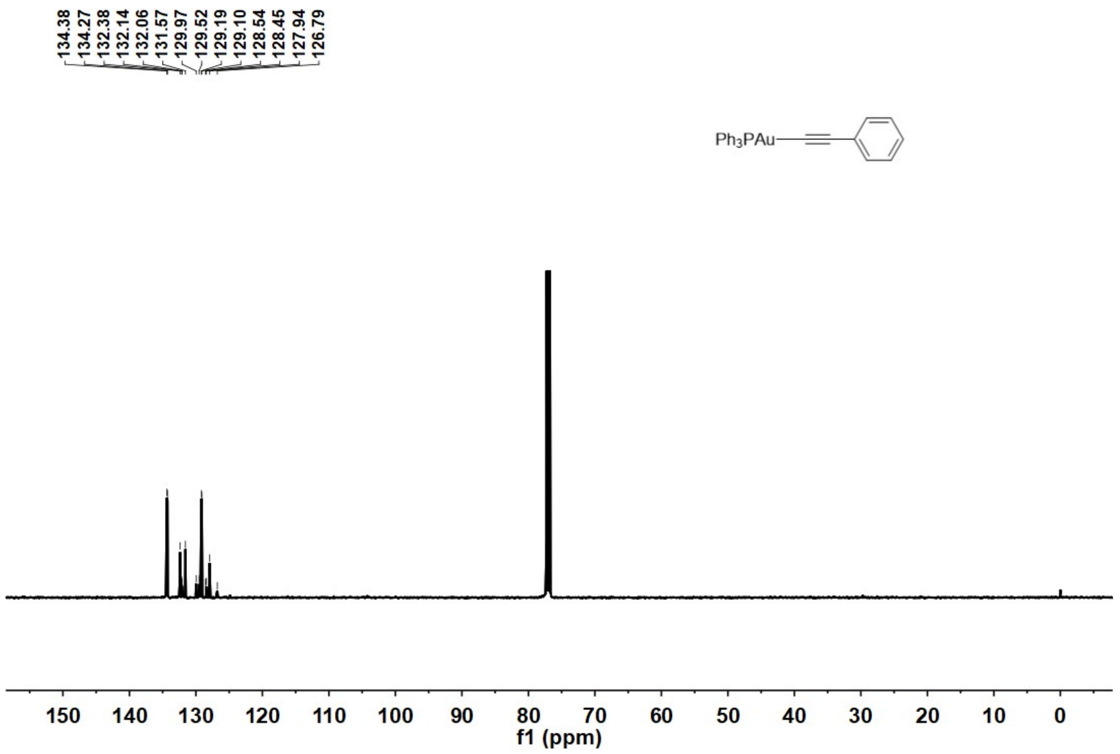


**Fig. S2** $^{13}C$ NMR spectra of complex **1a** in $CDCl_3$

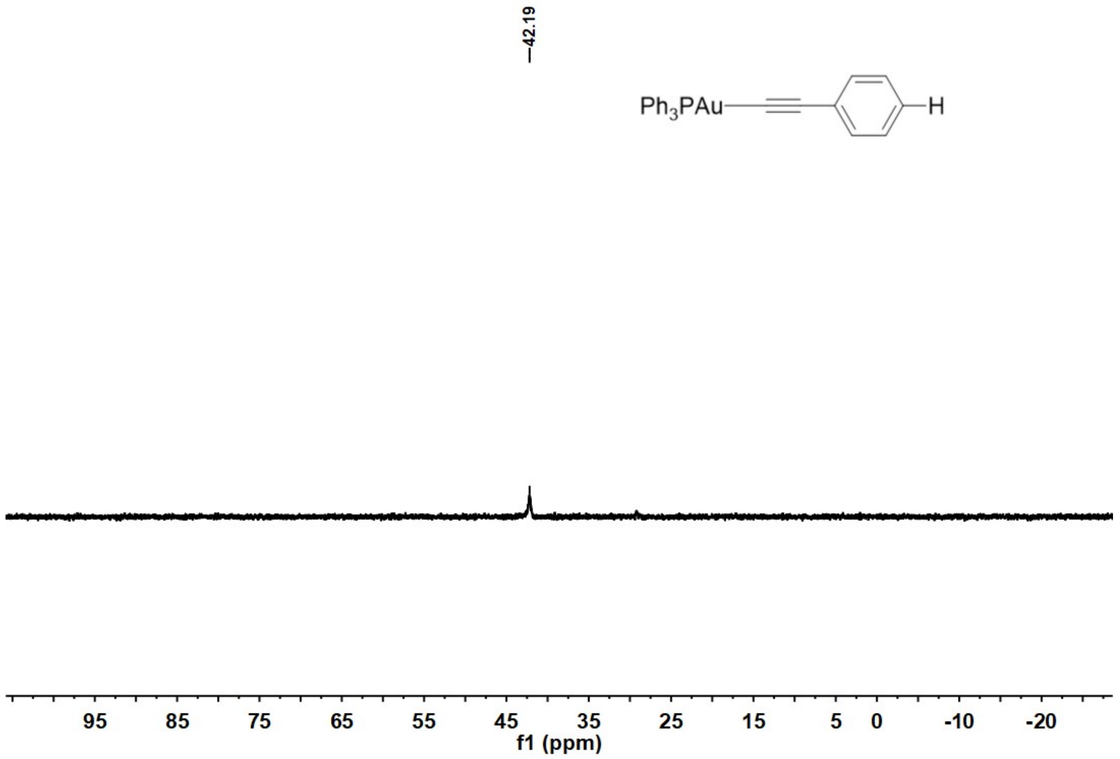


**Fig. S3** $^{31}P$ NMR spectra of complex **1a** in $CDCl_3$

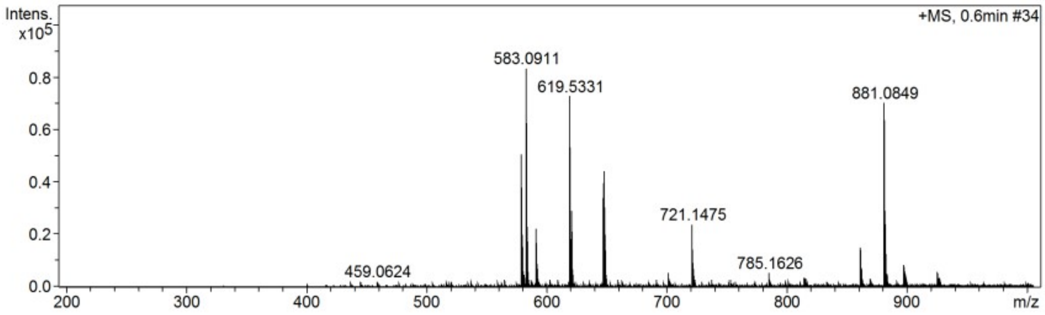


**Fig. S4.** MS spectra of complex **1a**.

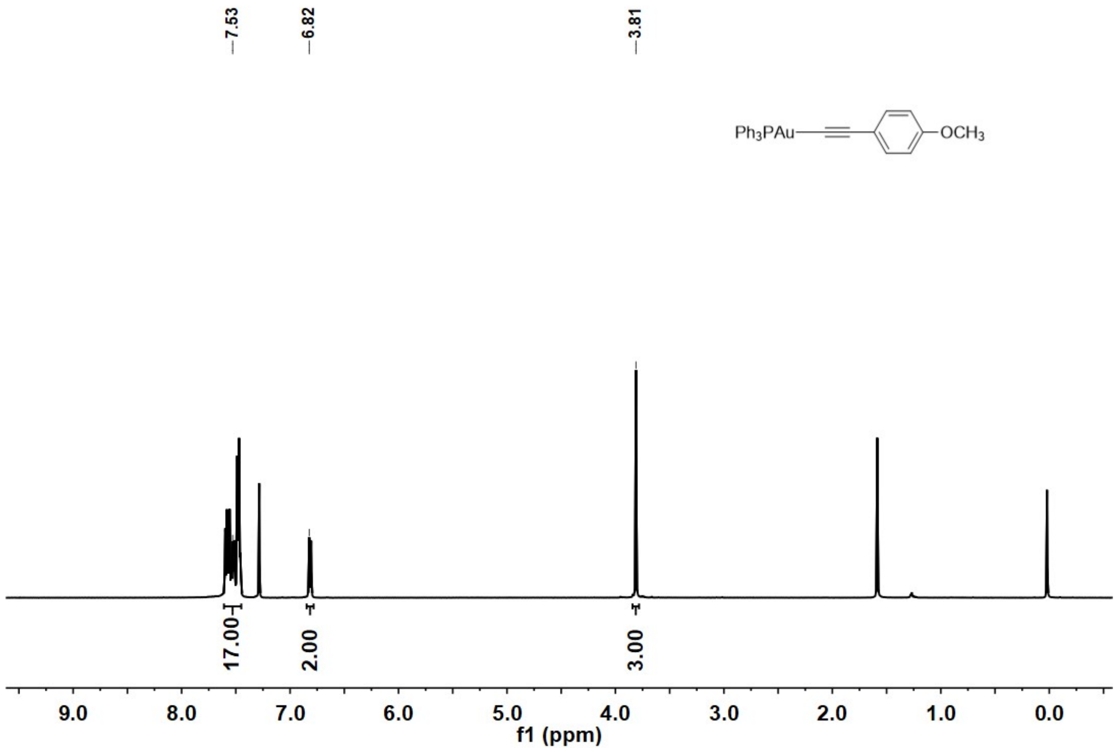


**Fig. S5** $^{1}$H NMR spectra of complex **1b** in $CDCl_3$

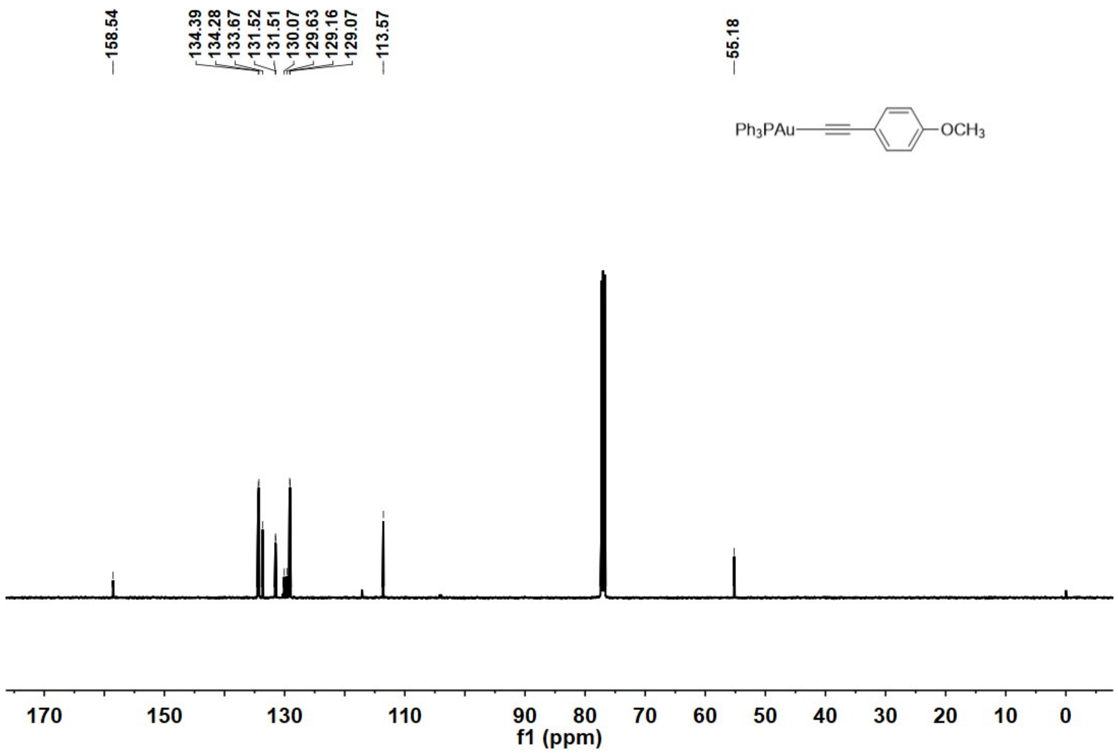


**Fig. S6** $^{13}$C NMR spectra of complex **1b** in $CDCl_3$

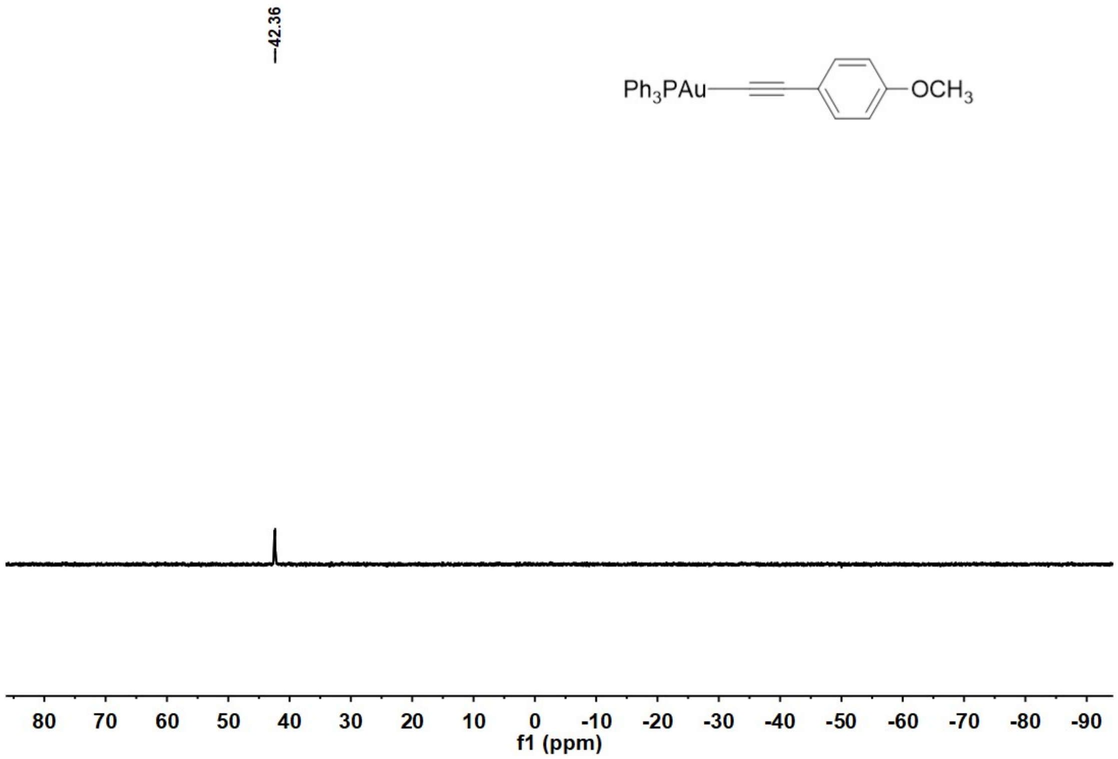


**Fig. S7** $^{31}$P NMR spectra of complex **1b** in $CDCl_3$

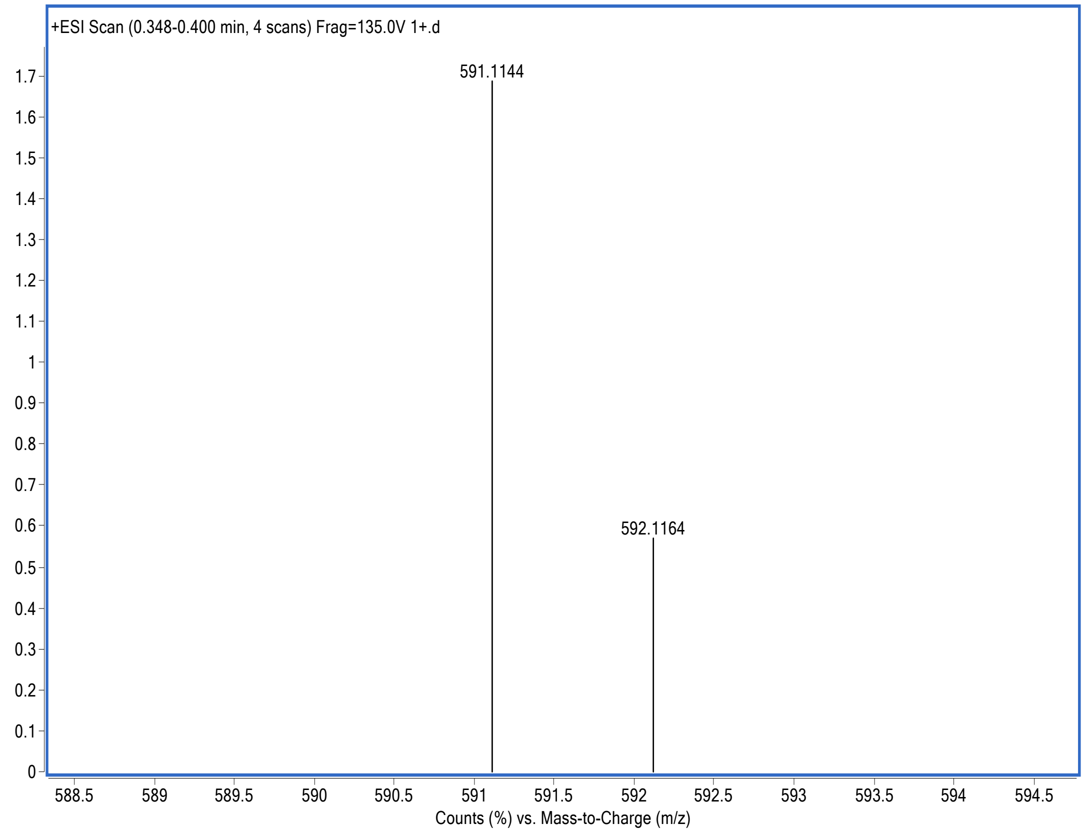


**Fig. S8.** MS spectra of complex **1b**.

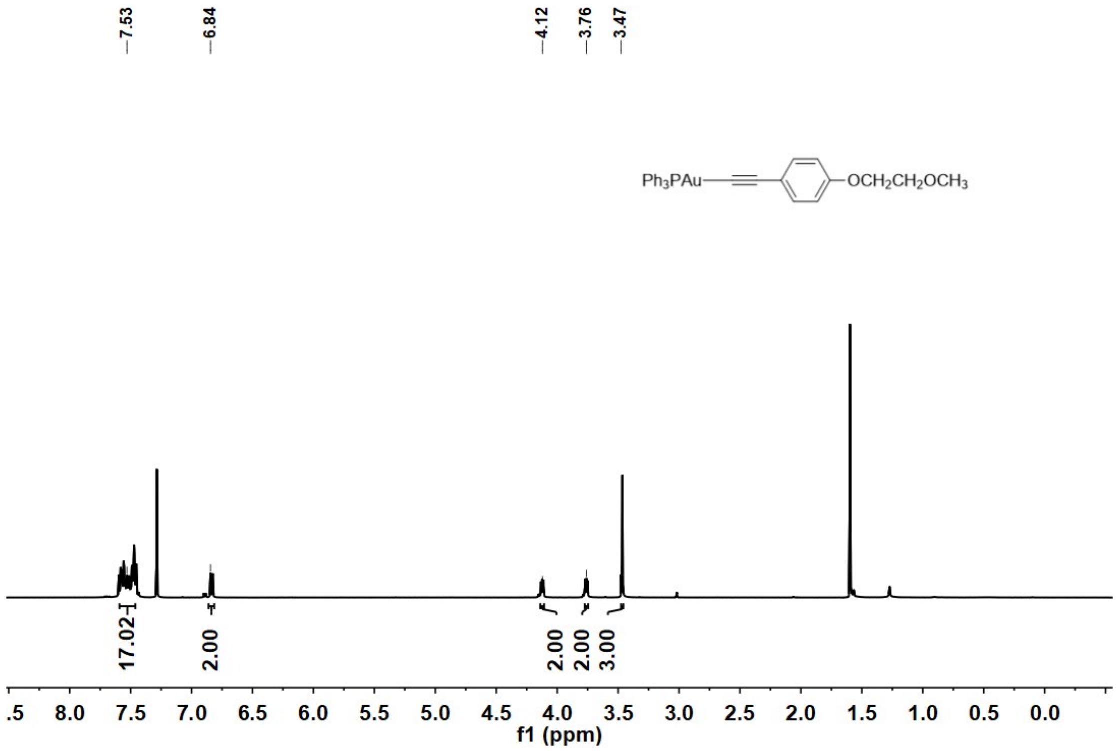


**Fig. S9** $^{1}H$ NMR spectra of complex **1c** in $CDCl_3$

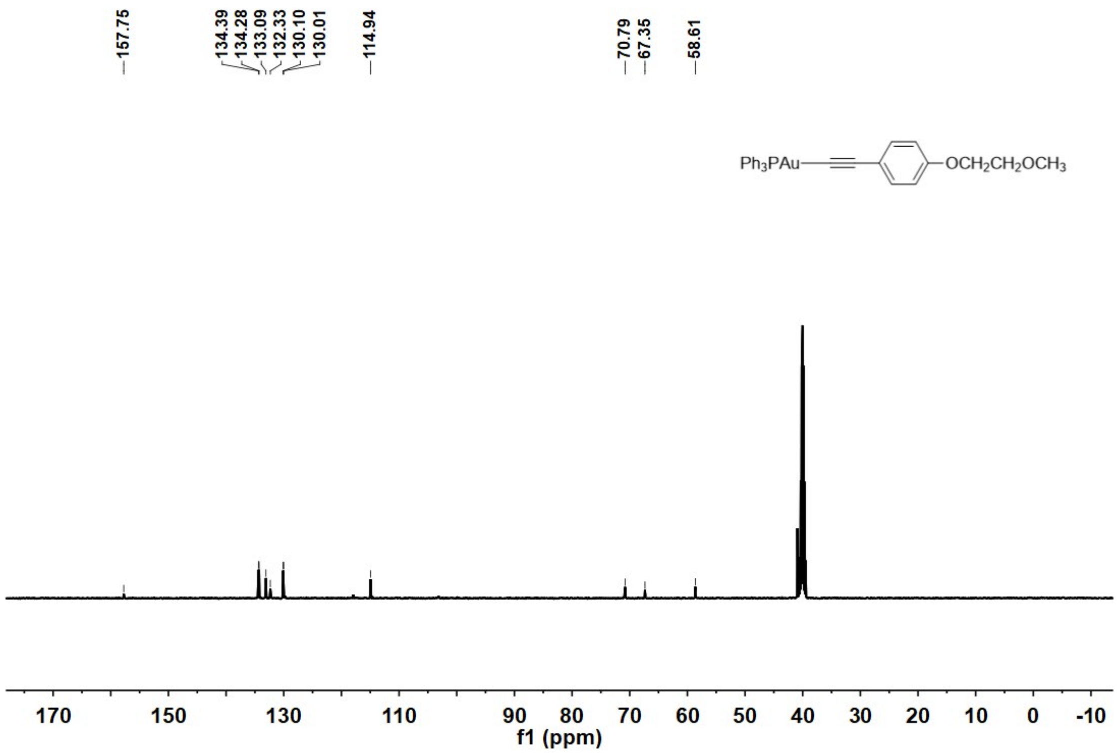


**Fig. S10** $^{13}C$ NMR spectra of complex **1c** in DMSO

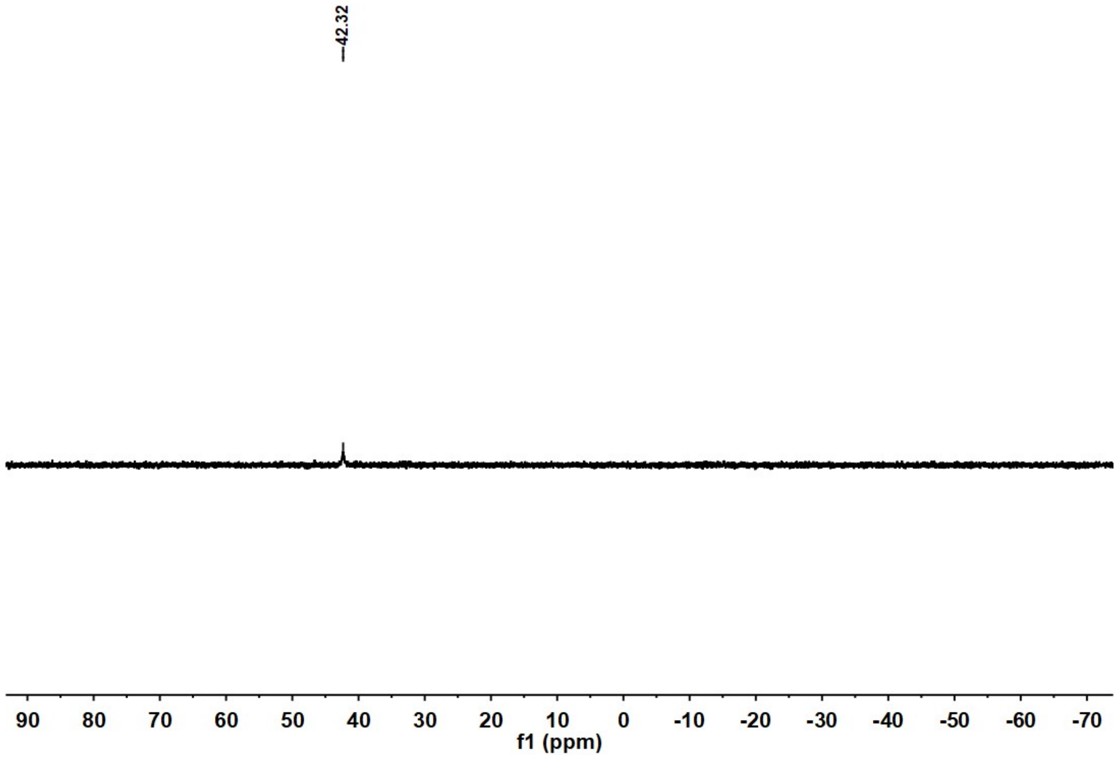


**Fig. S11** $^{31}P$ NMR spectra of complex **1c** in $CDCl_3$

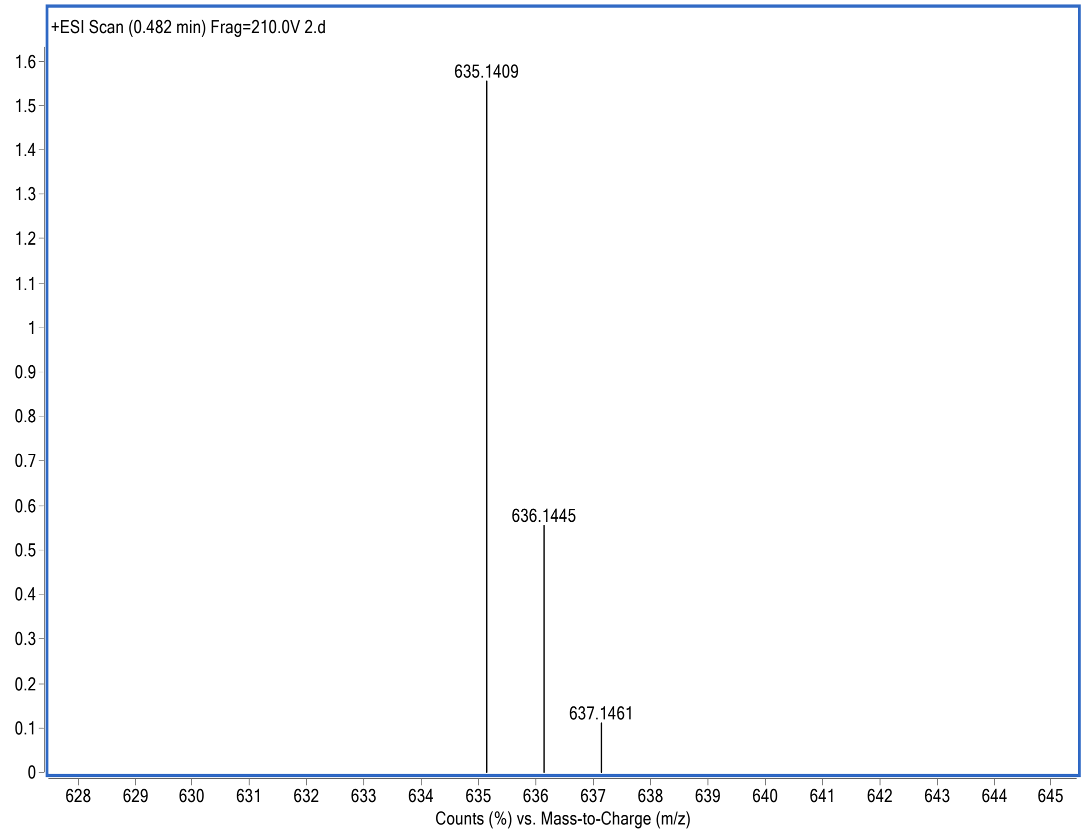


**Fig. S12.** MS spectra of complex **1c**.

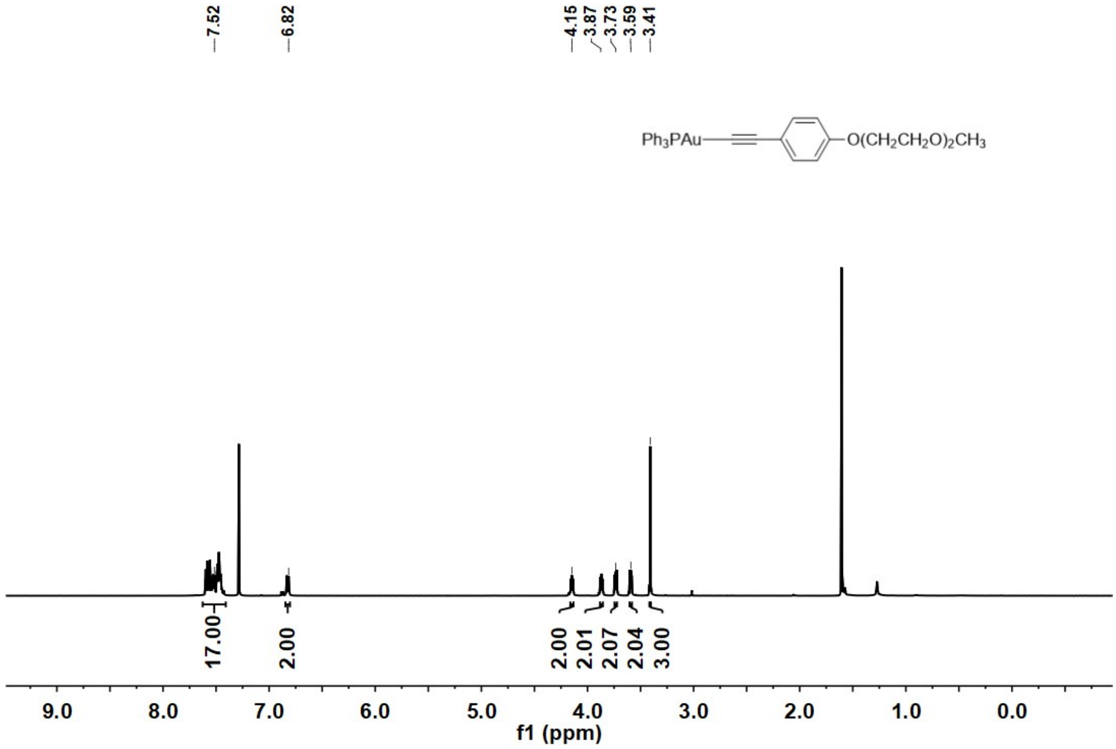


**Fig. S13** $^1H$ NMR spectra of complex **1d** in $CDCl_3$

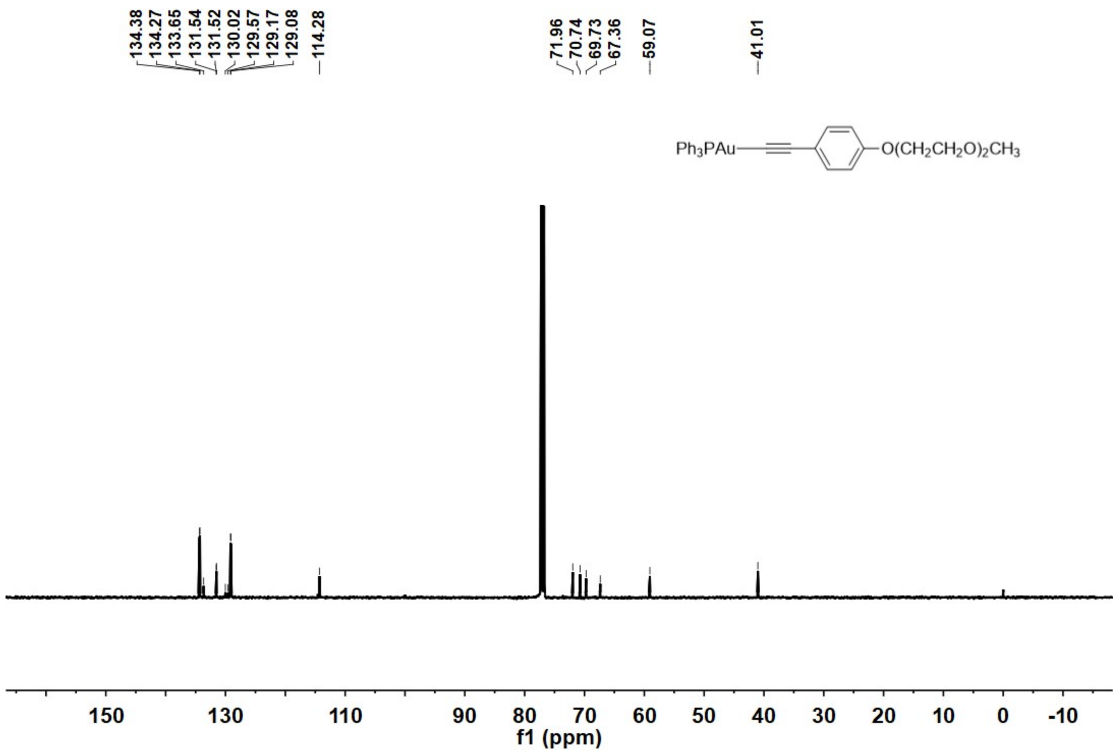


**Fig. S14** $^{13}$C NMR spectra of complex **1d** in $CDCl_3$

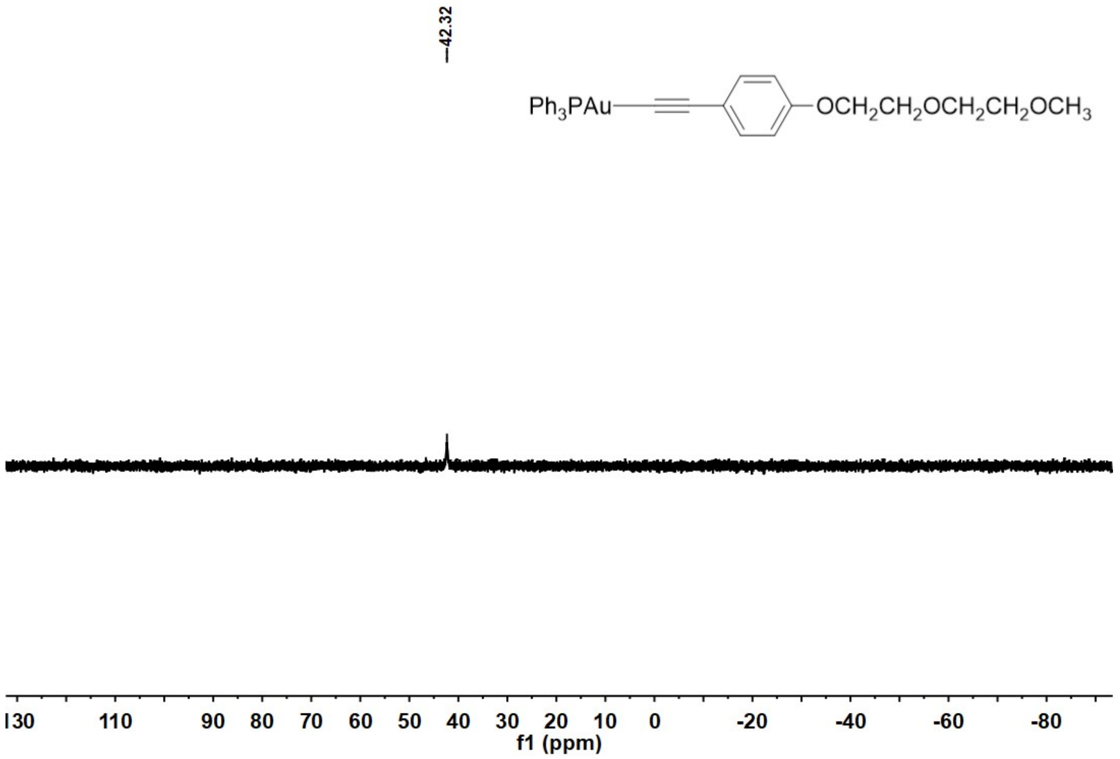


**Fig. S15** $^{31}$P NMR spectra of complex **1d** in $CDCl_3$

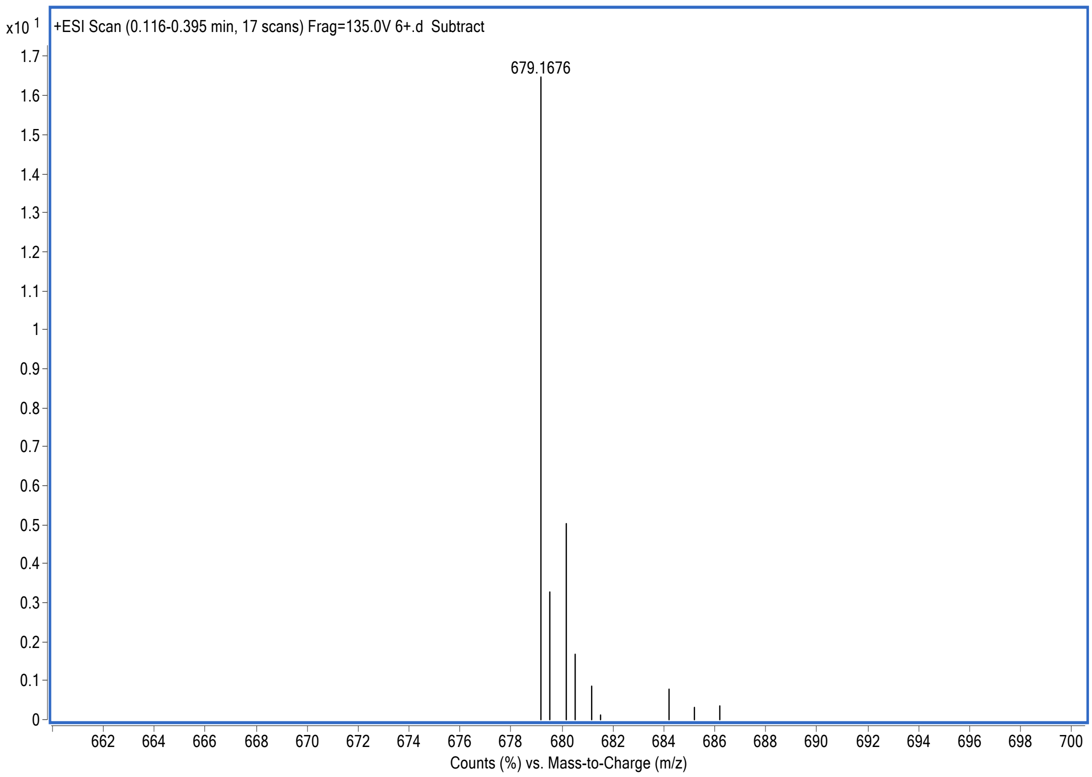


**Fig. S16.** MS spectra of complex **1d**.

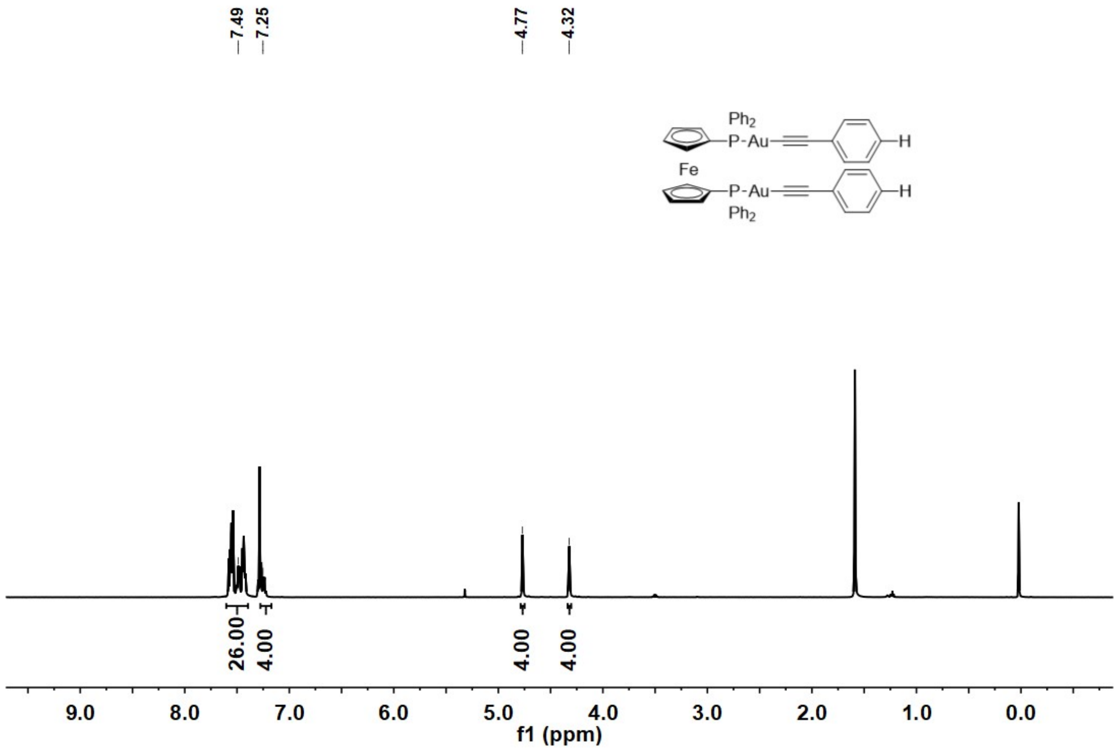


**Fig. S17** $^{1}$H NMR spectra of complex **2a** in $CDCl_3$

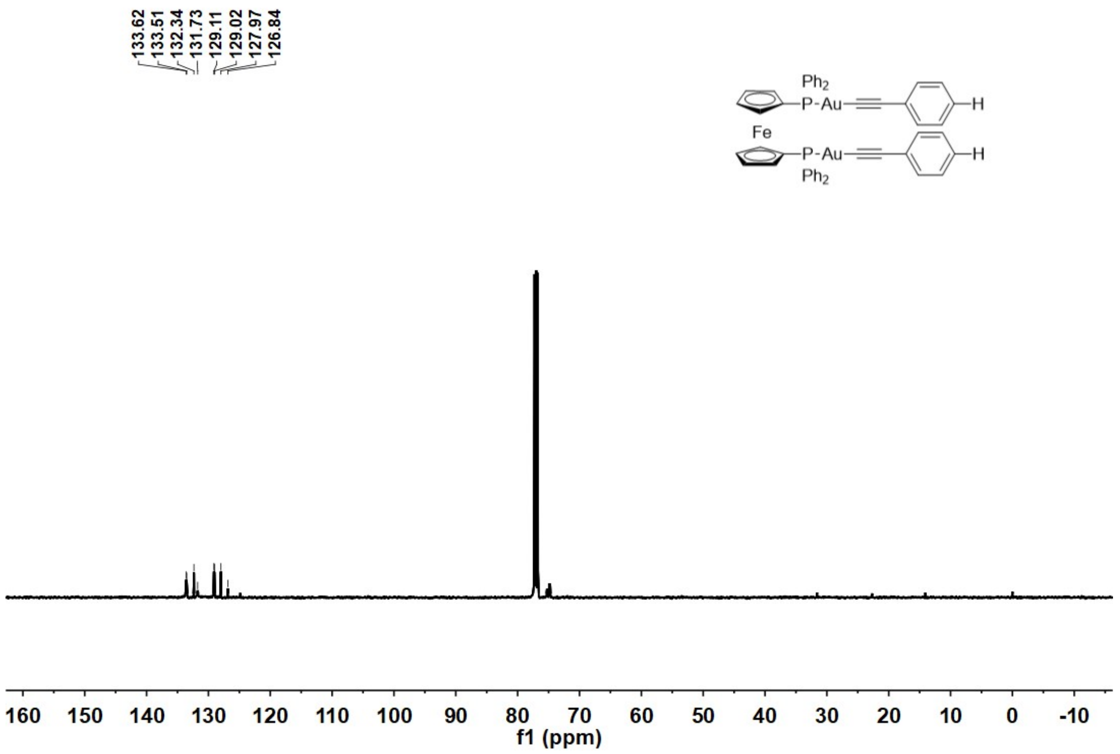


**Fig. S18** $^{13}C$ NMR spectra of complex **2a** in $CDCl_3$

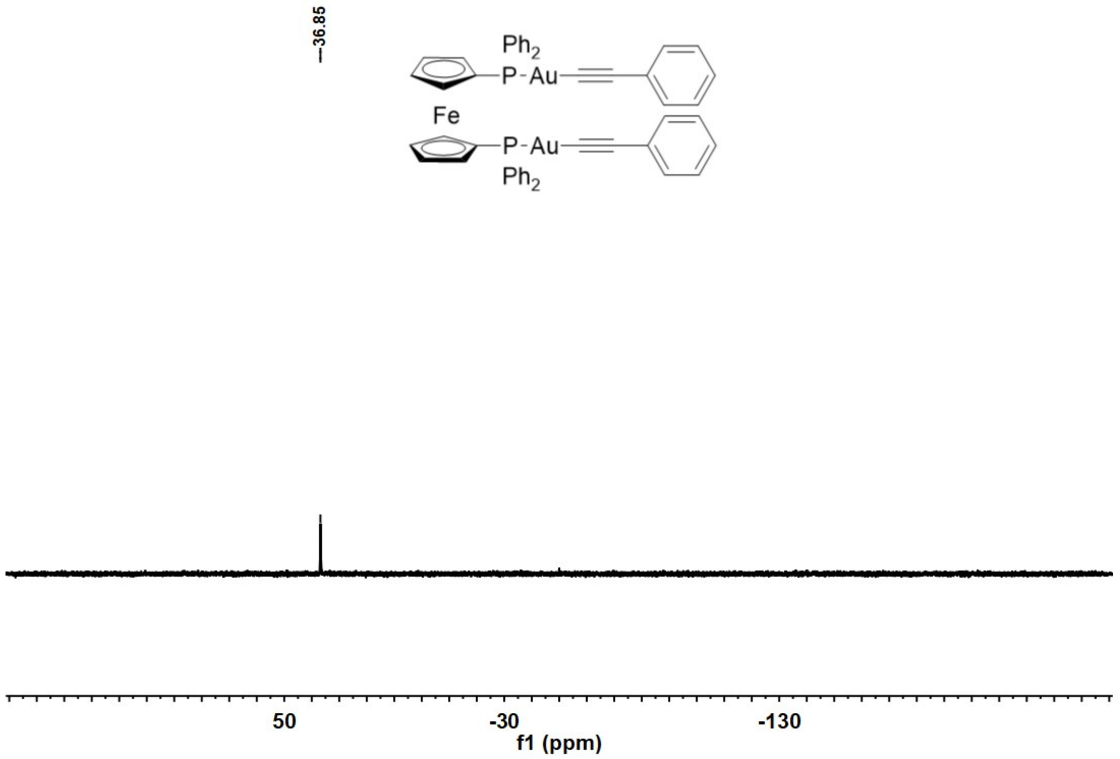


**Fig. S19** $^{31}P$ NMR spectra of complex **2a** in $CDCl_3$

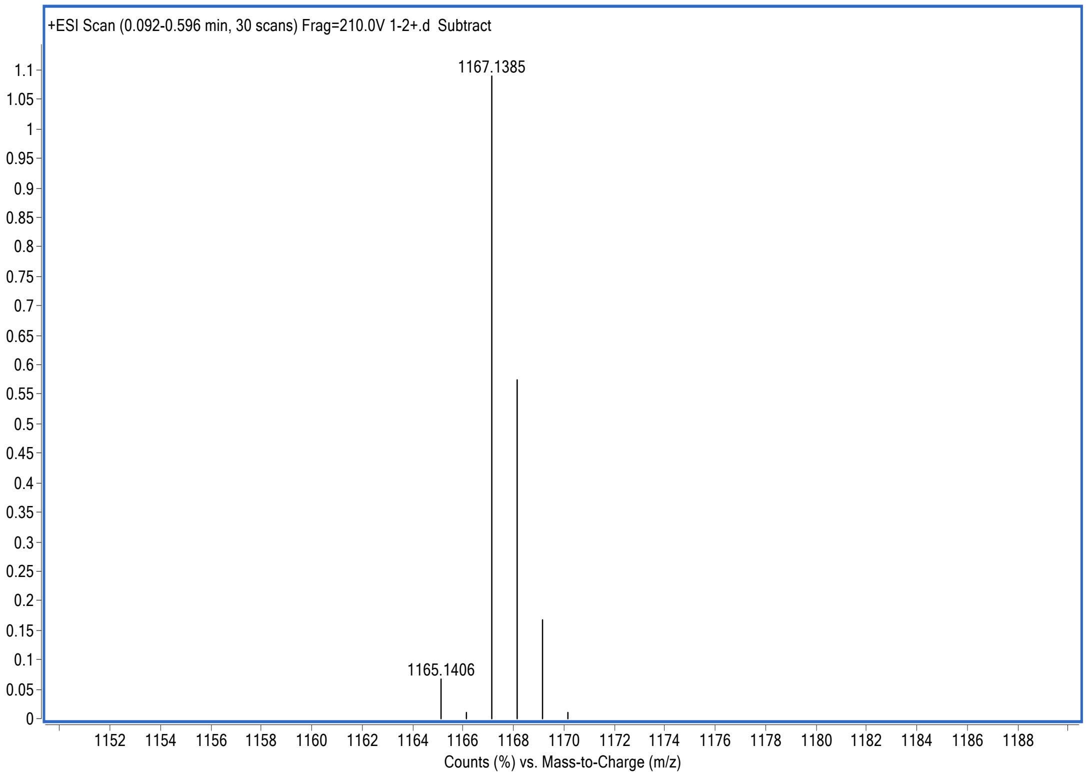


**Fig. S20.** MS spectra of complex **2a**.

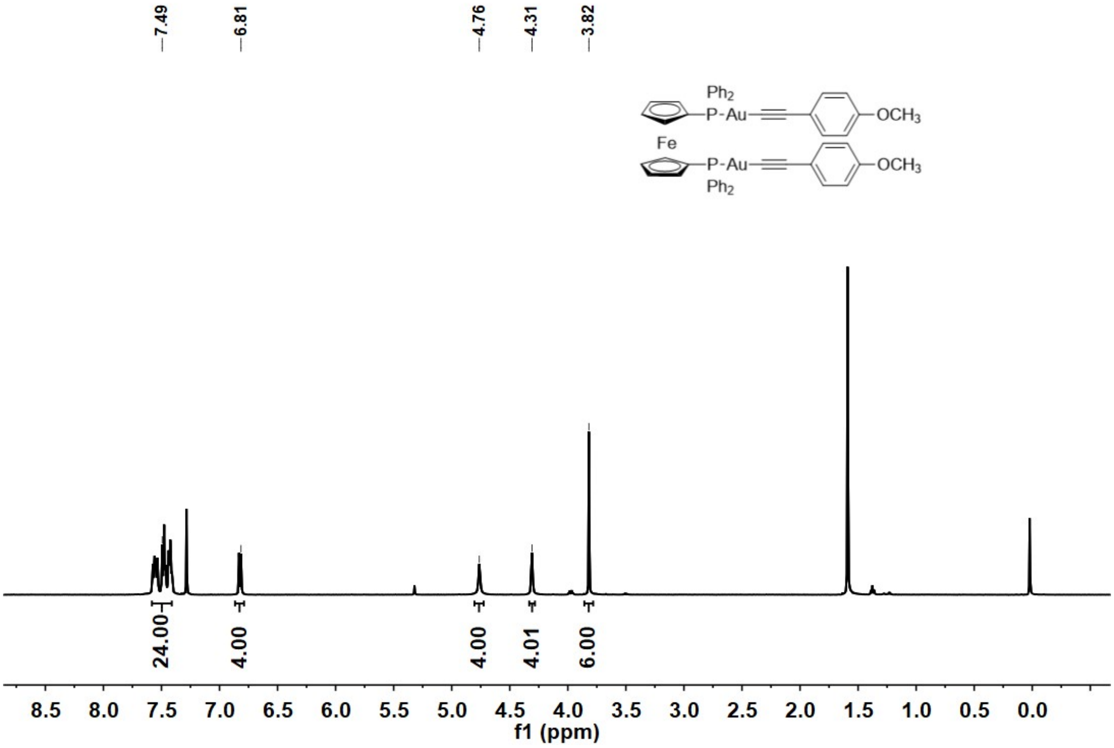


**Fig. S21** $^{1}$H NMR spectra of complex **2b** in $CDCl_3$

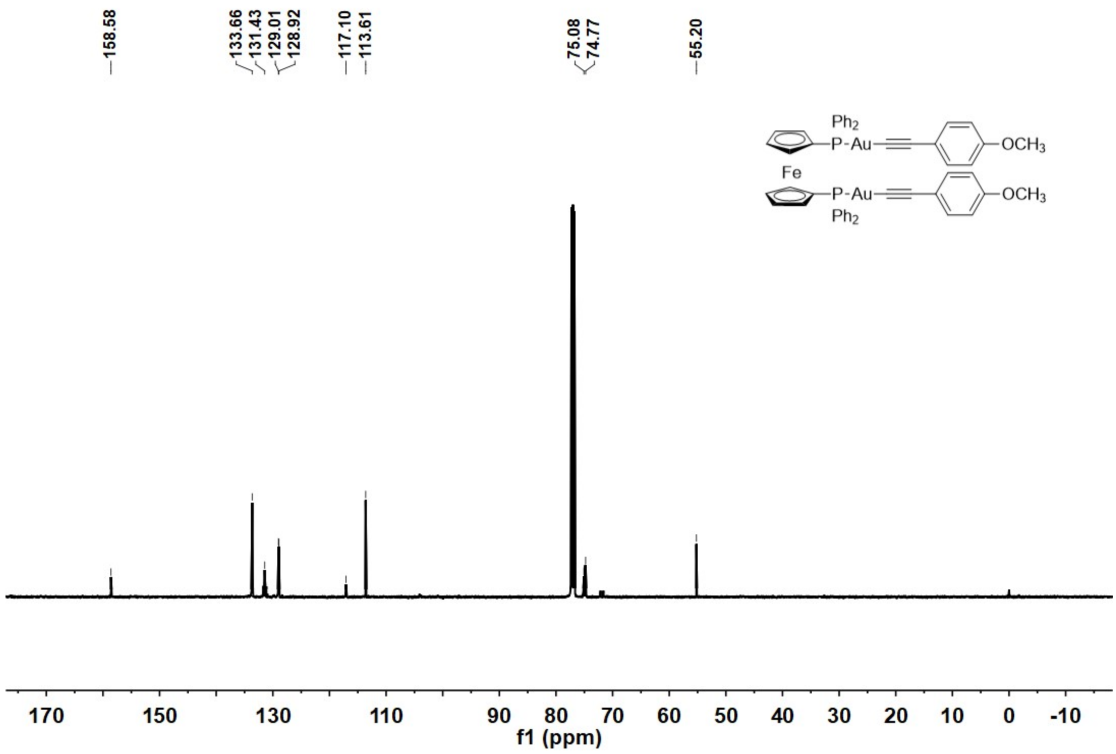


**Fig. S22** $^{13}C$ NMR spectra of complex **2b** in $CDCl_3$

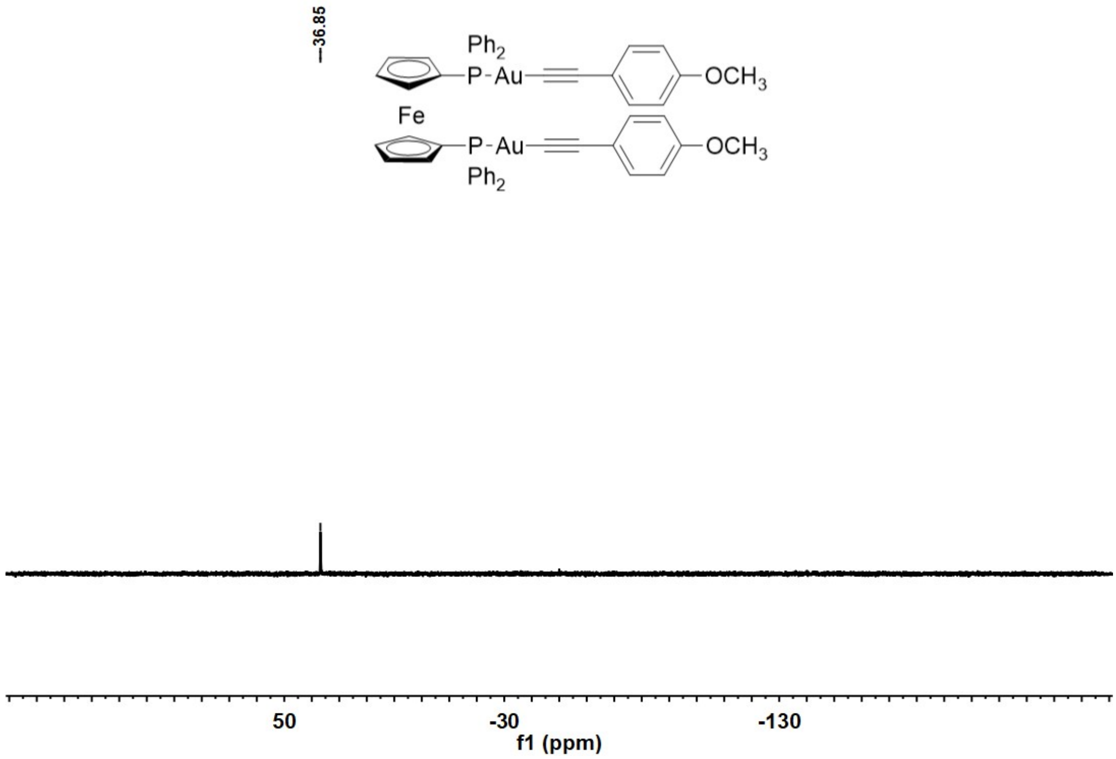


**Fig. S23** $^{31}P$ NMR spectra of complex **2b** in $CDCl_3$

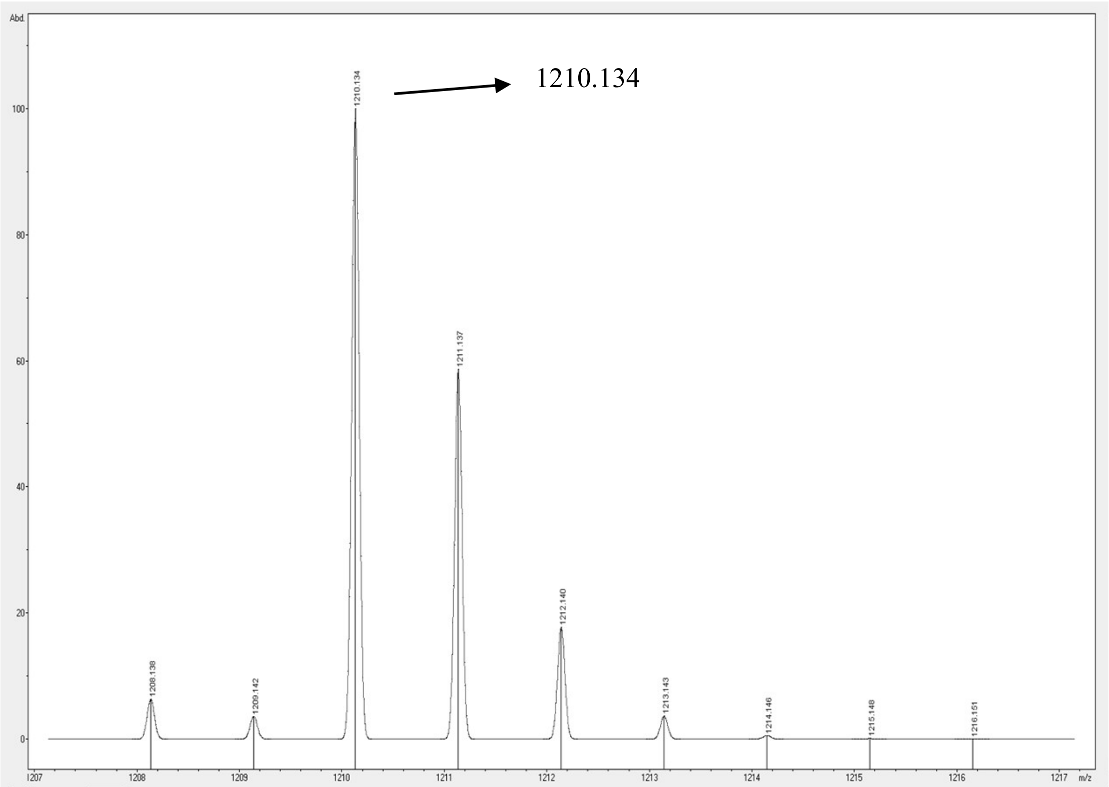


**Fig. S24.** MS spectra of complex **2b**.

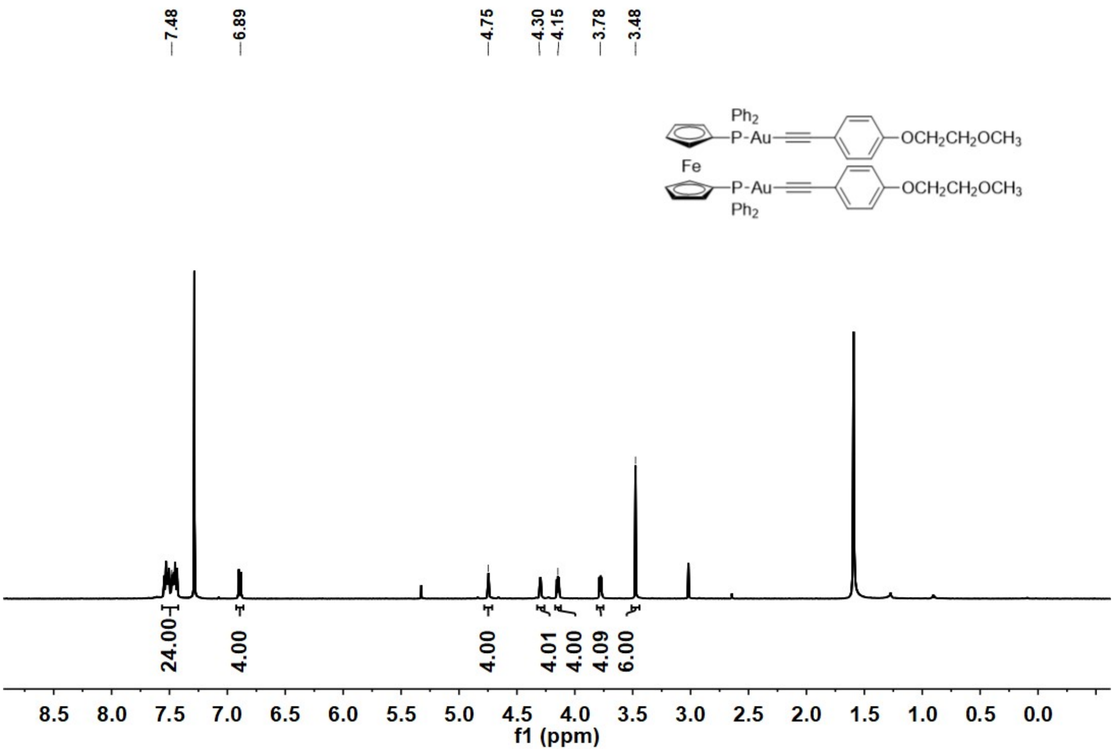


**Fig. S25** $^{1}H$ NMR spectra of complex **2c** in $CDCl_3$

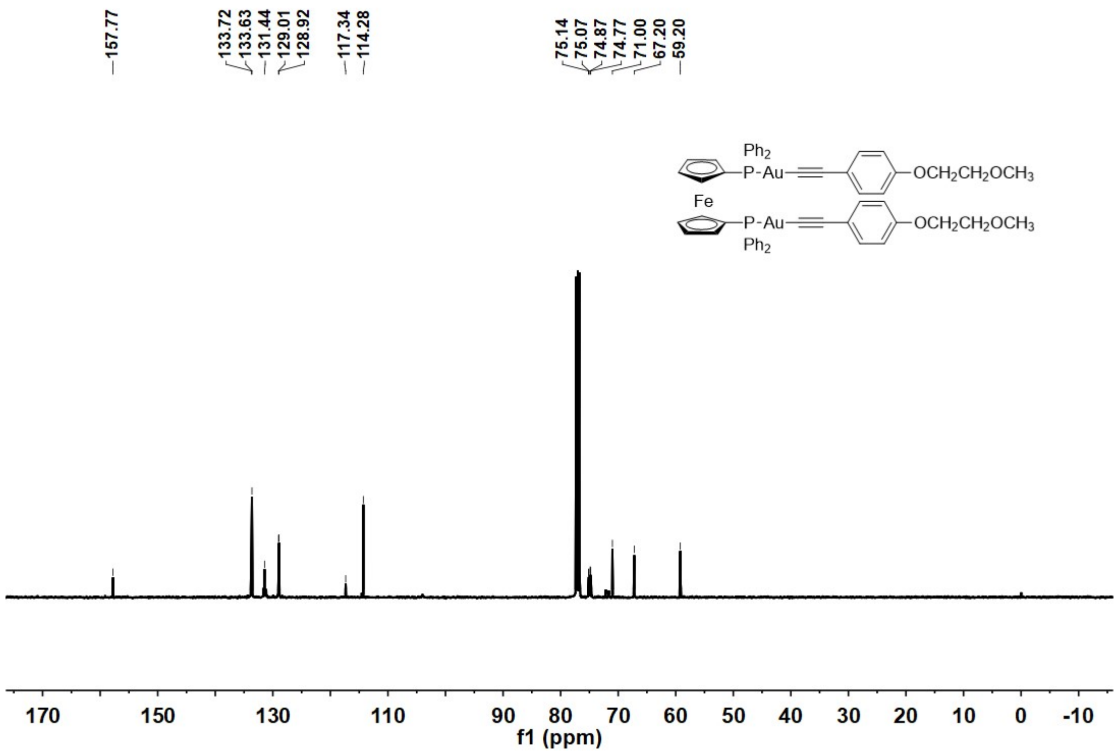


**Fig. S26** $^{13}C$ NMR spectra of complex **2c** in $CDCl_3$

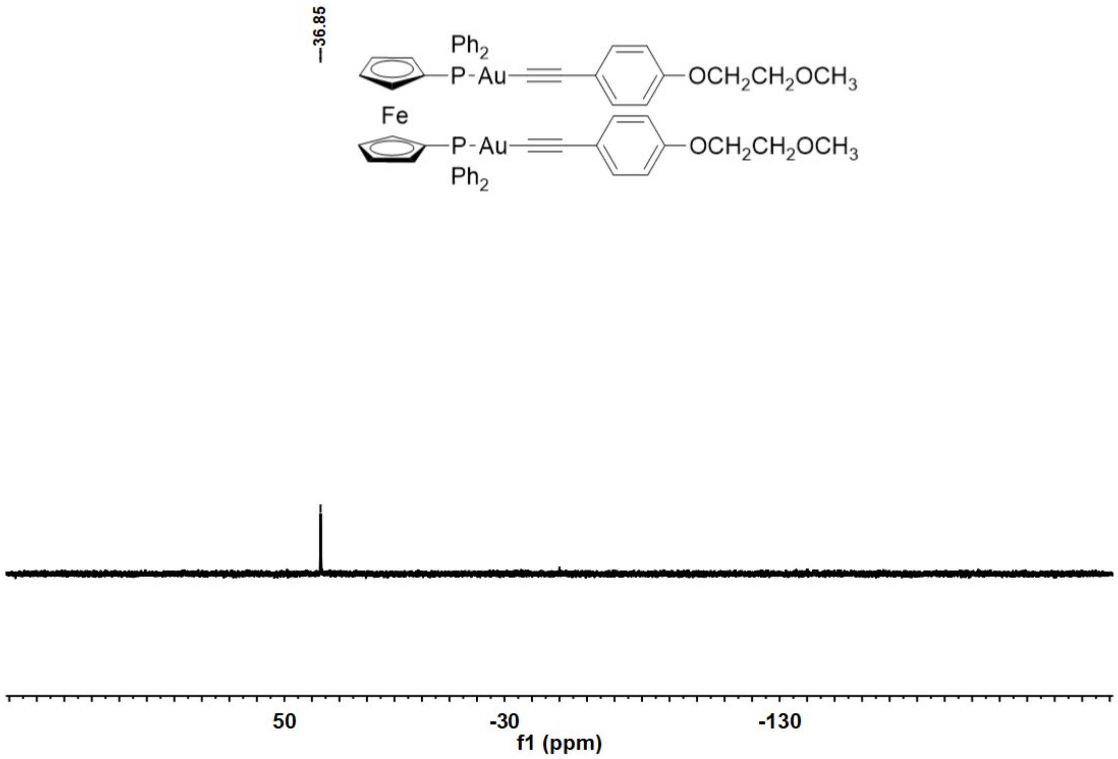


**Fig. S27** $^{31}P$ NMR spectra of complex **2c** in $CDCl_3$

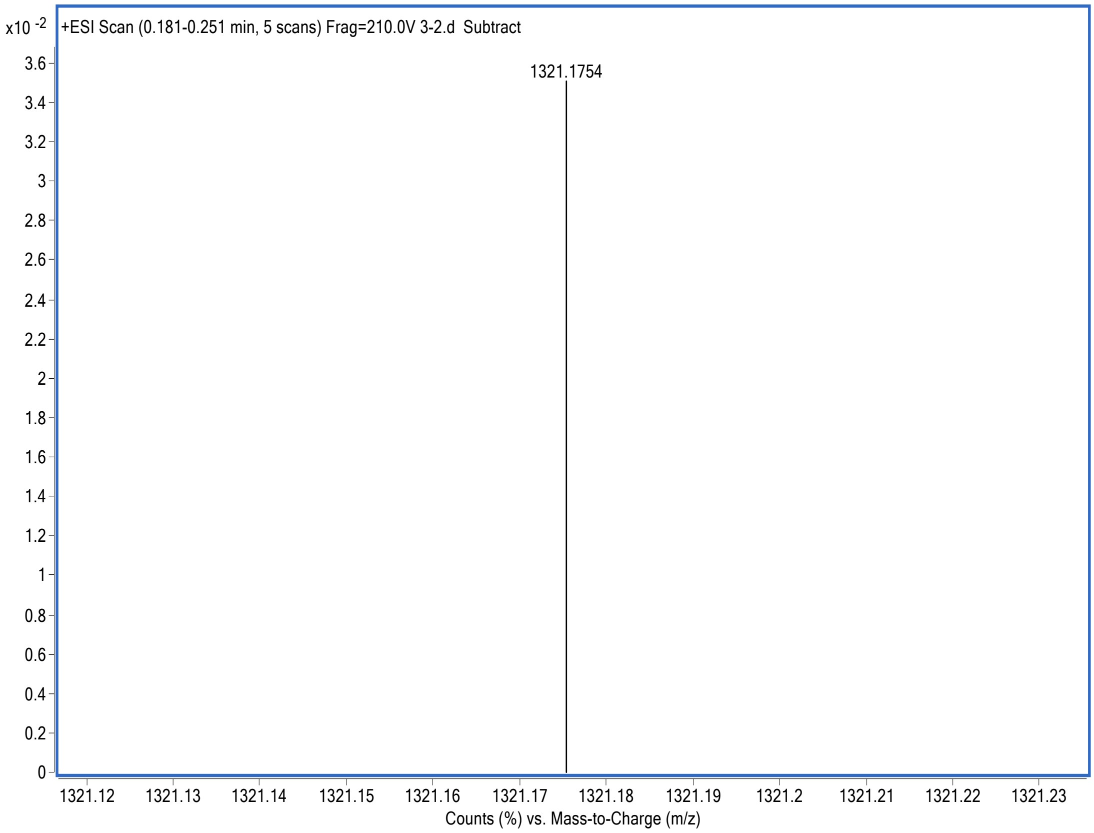


**Fig. S28.** MS spectra of complex **2b**.

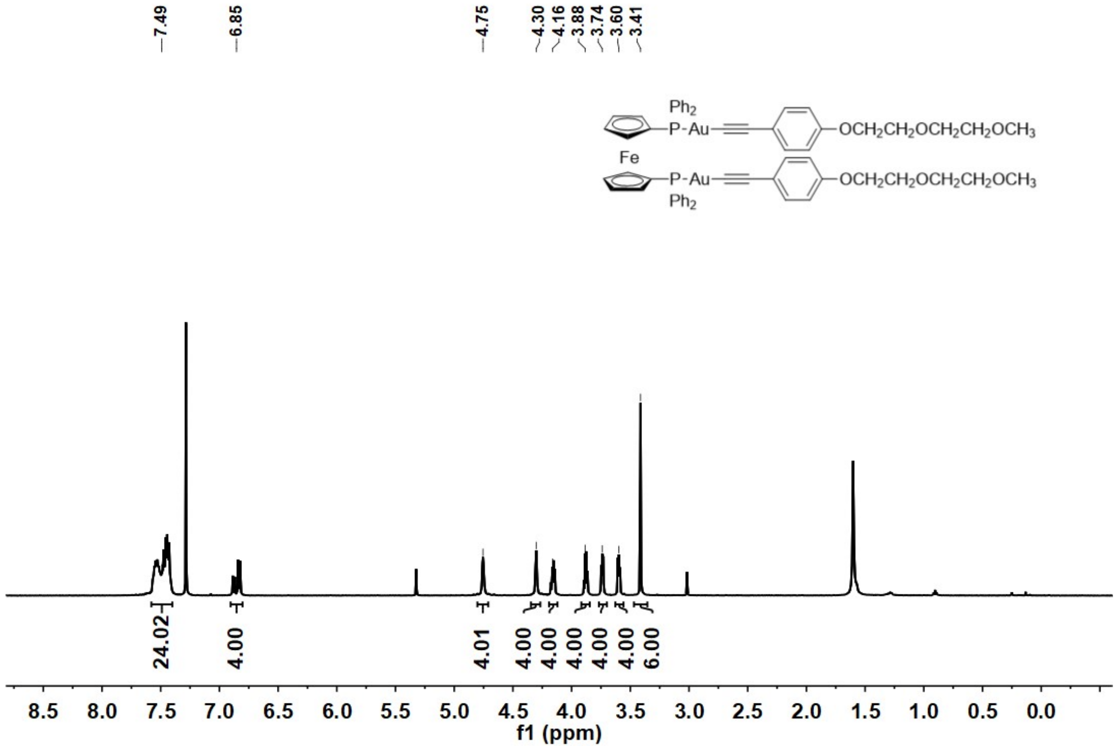


**Fig. S29** $^{1}H$ NMR spectra of complex **2d** in $CDCl_3$

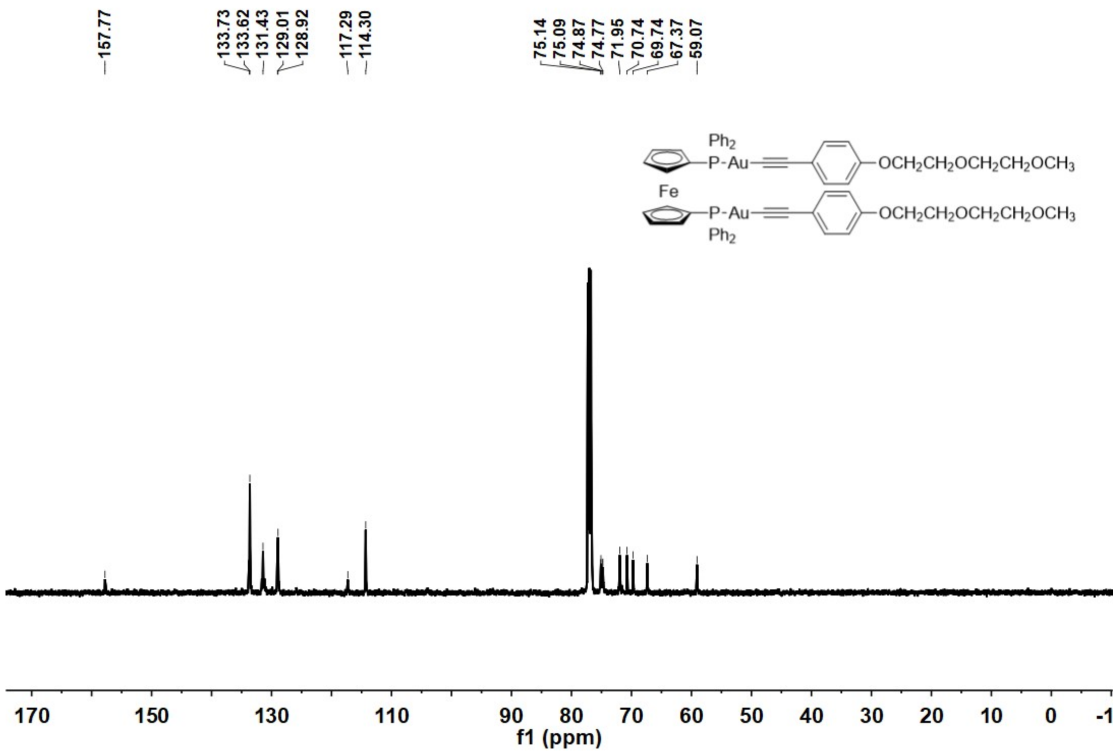


**Fig. S30** $^{13}C$ NMR spectra of complex **2d** in $CDCl_3$

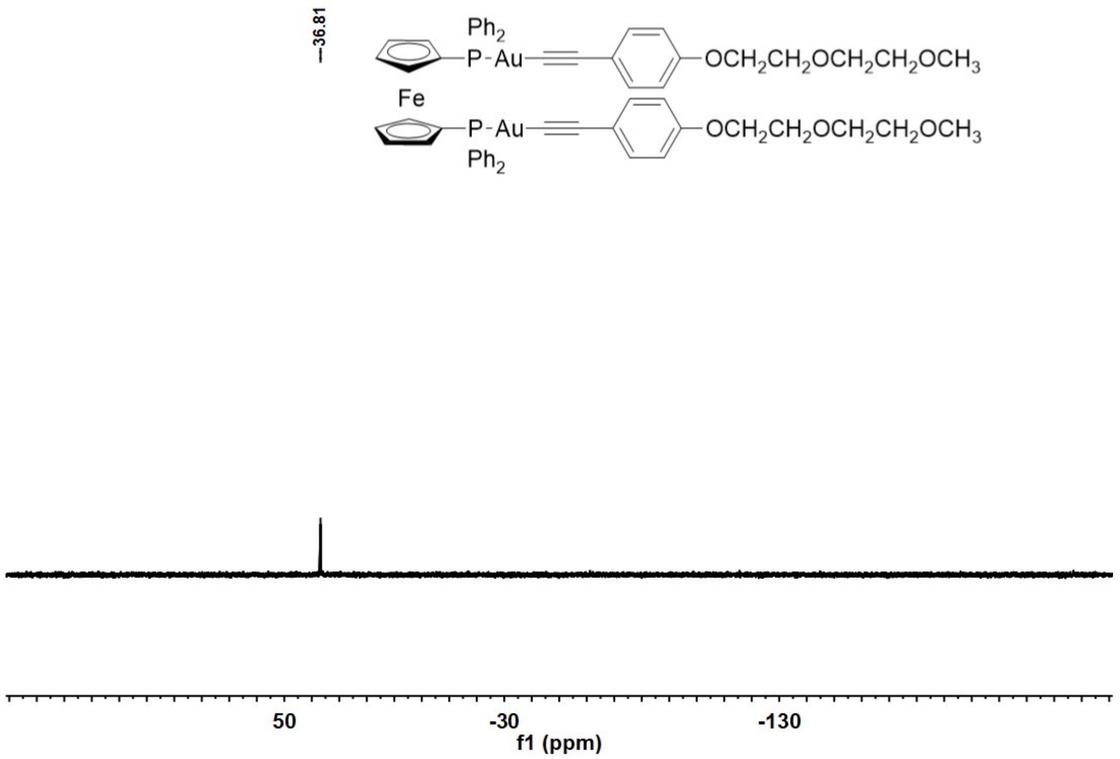


**Fig. S31** $^{31}P$ NMR spectra of complex **2d** in $CDCl_3$

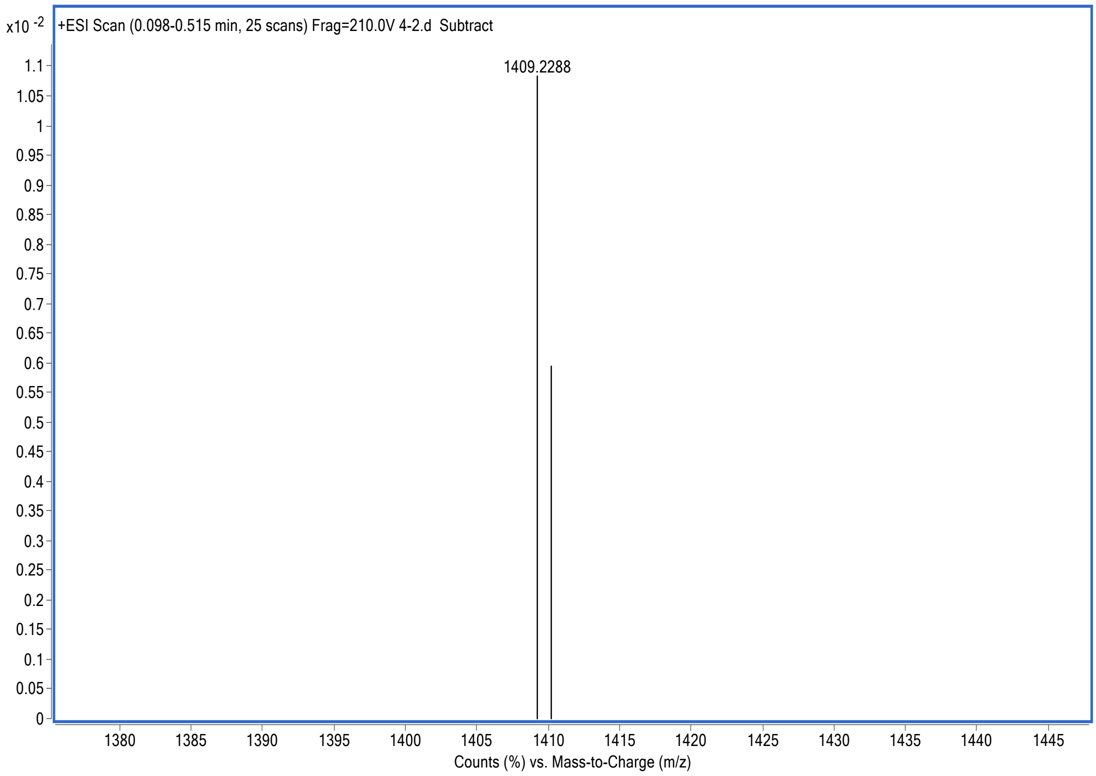


**Fig. S32** MS spectra of complex **2d**.

## 3. Antiproliferative activity

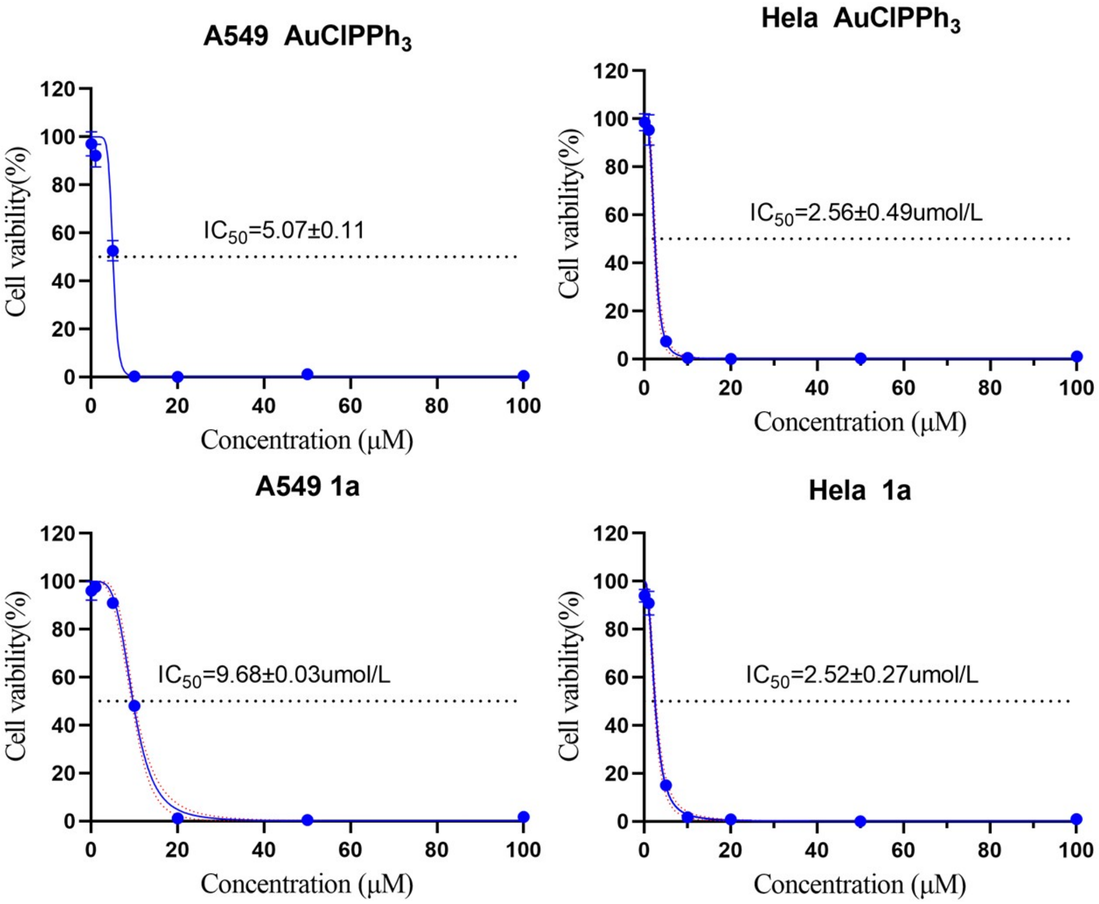

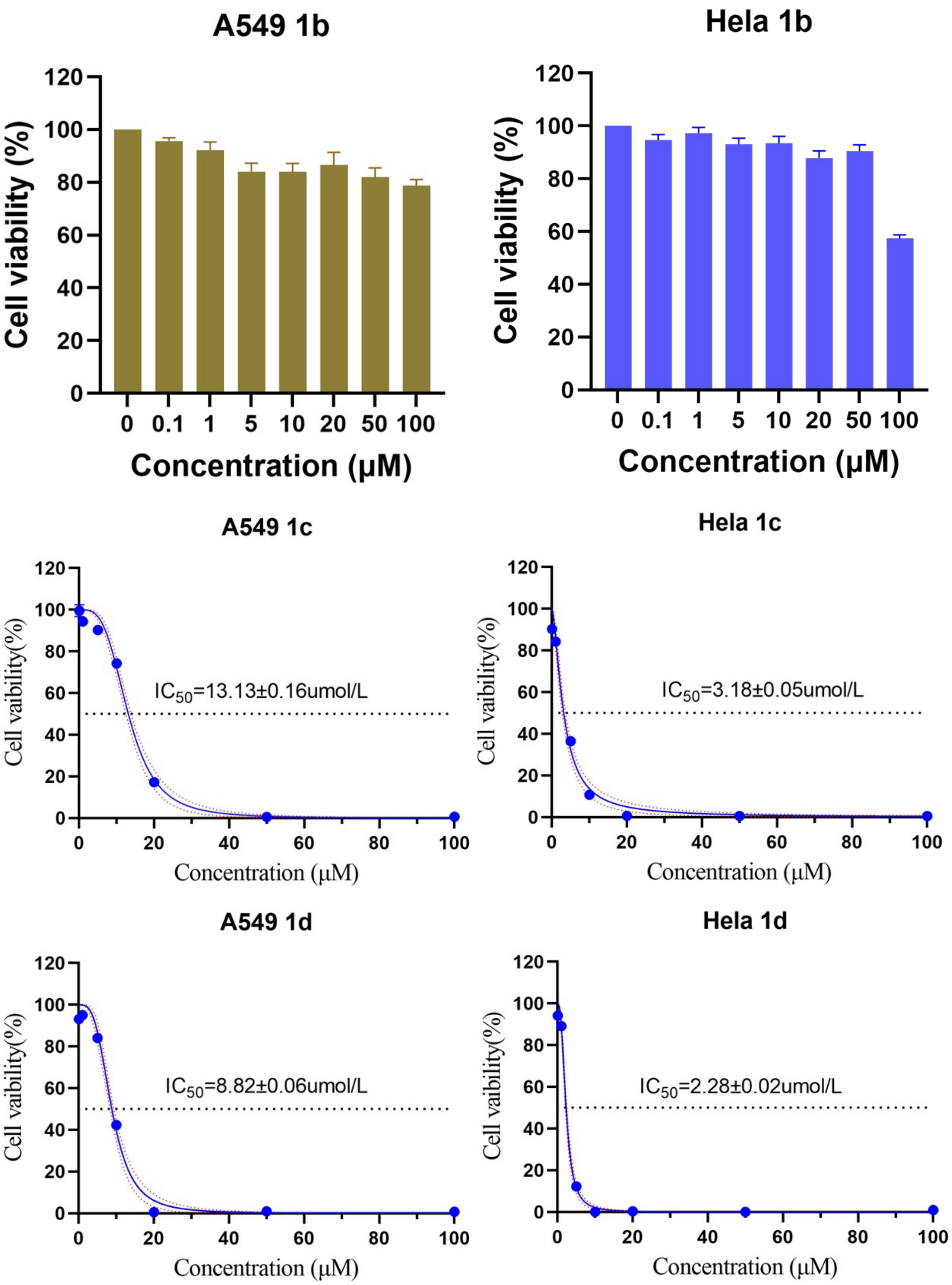
A549 1b
Hela 1b
Cell viability (%)
Concentration (μM)
A549 1c
IC50=13.13±0.16umol/L
Hela 1c
IC50=3.18±0.05umol/L
A549 1d
IC50=8.82±0.06umol/L
Hela 1d
IC50=2.28±0.02umol/L
Cell vaibility(%)
Concentration (μM)

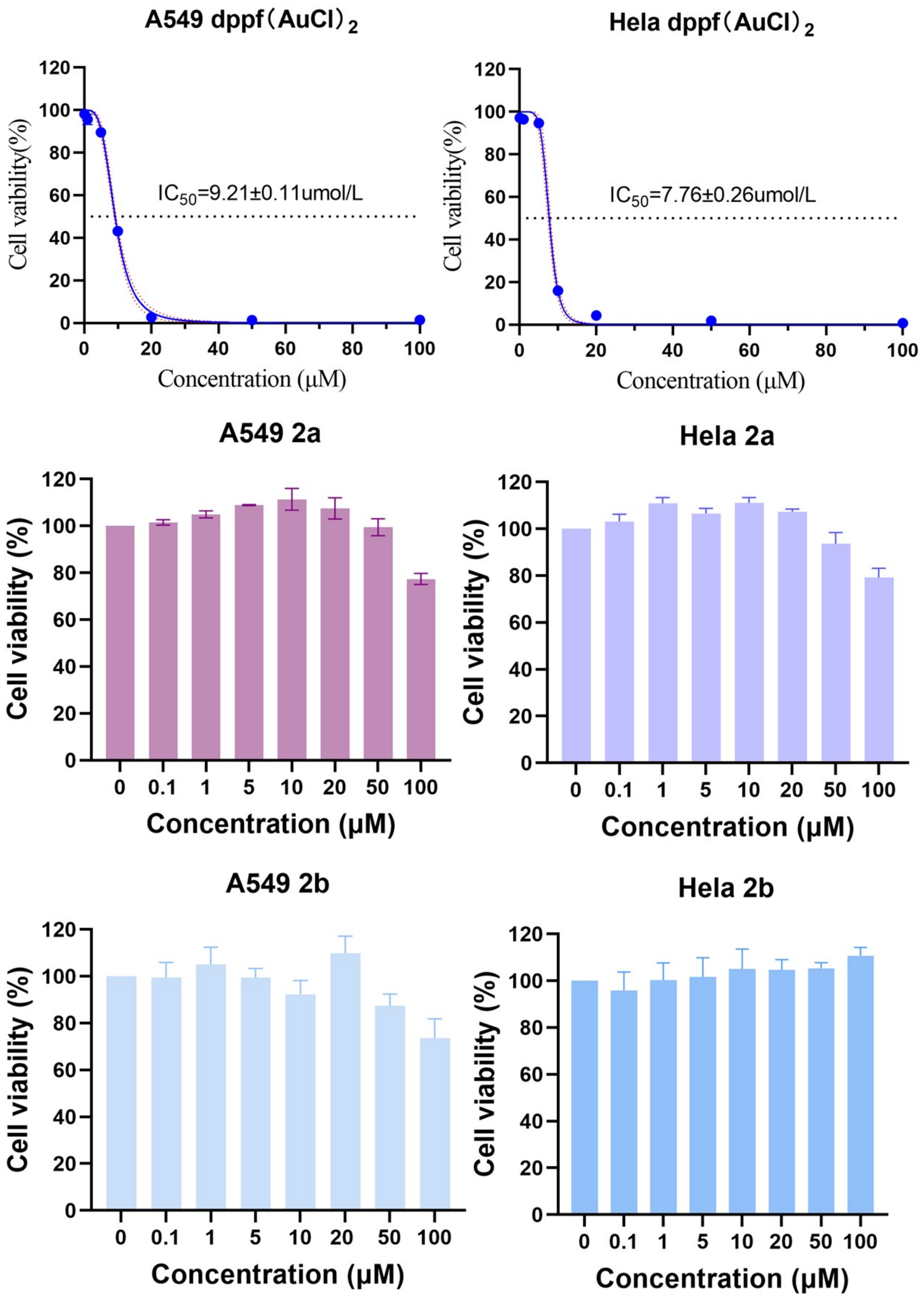
A549 dppf（AuCl）2
Hela dppf（AuCl）2
IC50=9.21±0.11umol/L
IC50=7.76±0.26umol/L
Cell vaibility(%)
Concentration (μM)
A549 2a
Hela 2a
A549 2b
Hela 2b
Cell viability (%)
Concentration (μM)
0 0.1 1 5 10 20 50 100

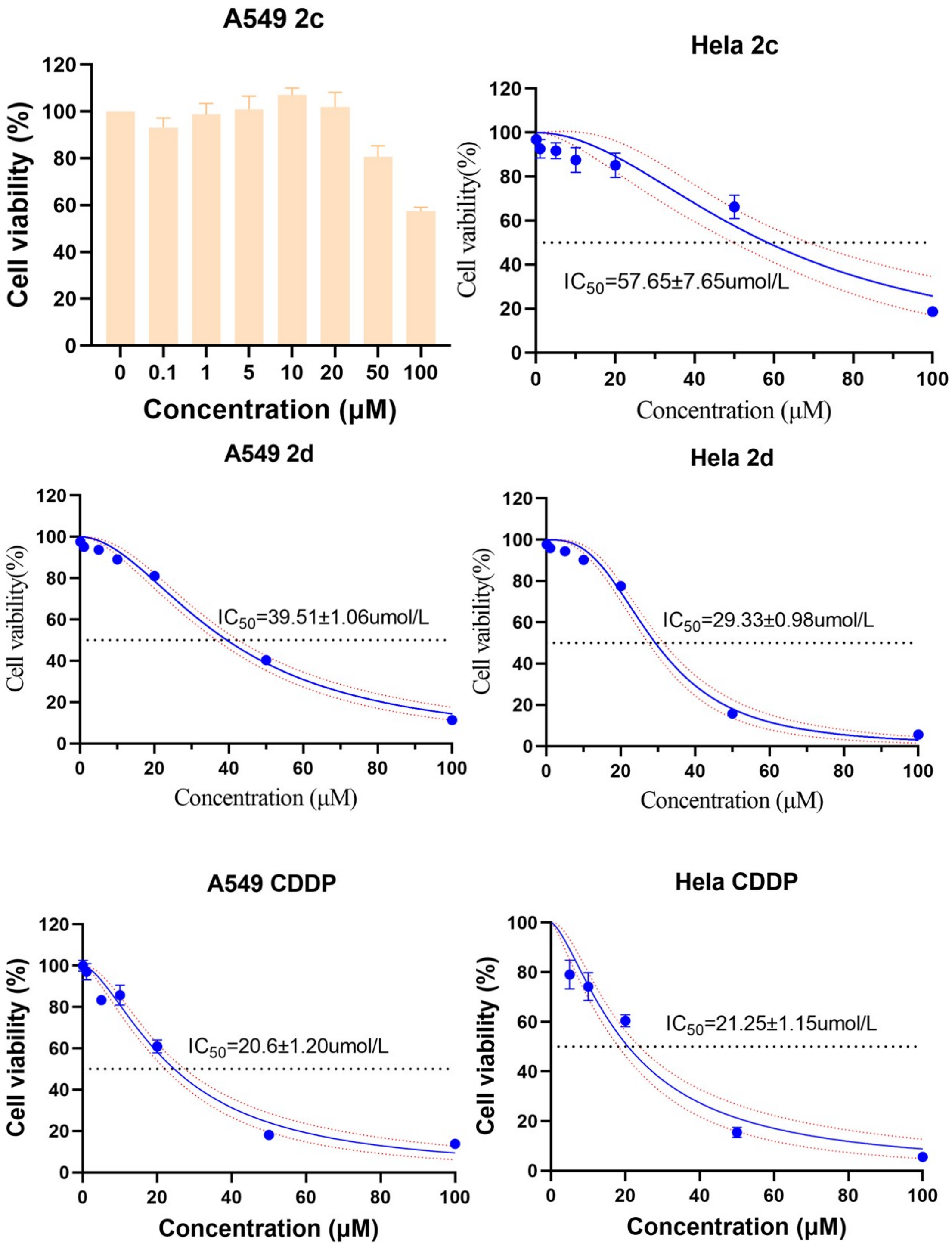


**Fig. S33** Antiproliferative activity of **$PPh_3AuCl$**, mono gold(I) complexes **1a~1d**, **dppf$(AuCl)_2$**, digold(I) complexes **2a~2d**, and cisplatin against A549 and Hela (24 h, CCK-8 assays, $IC_{50}$, mean ± SD [μM]).

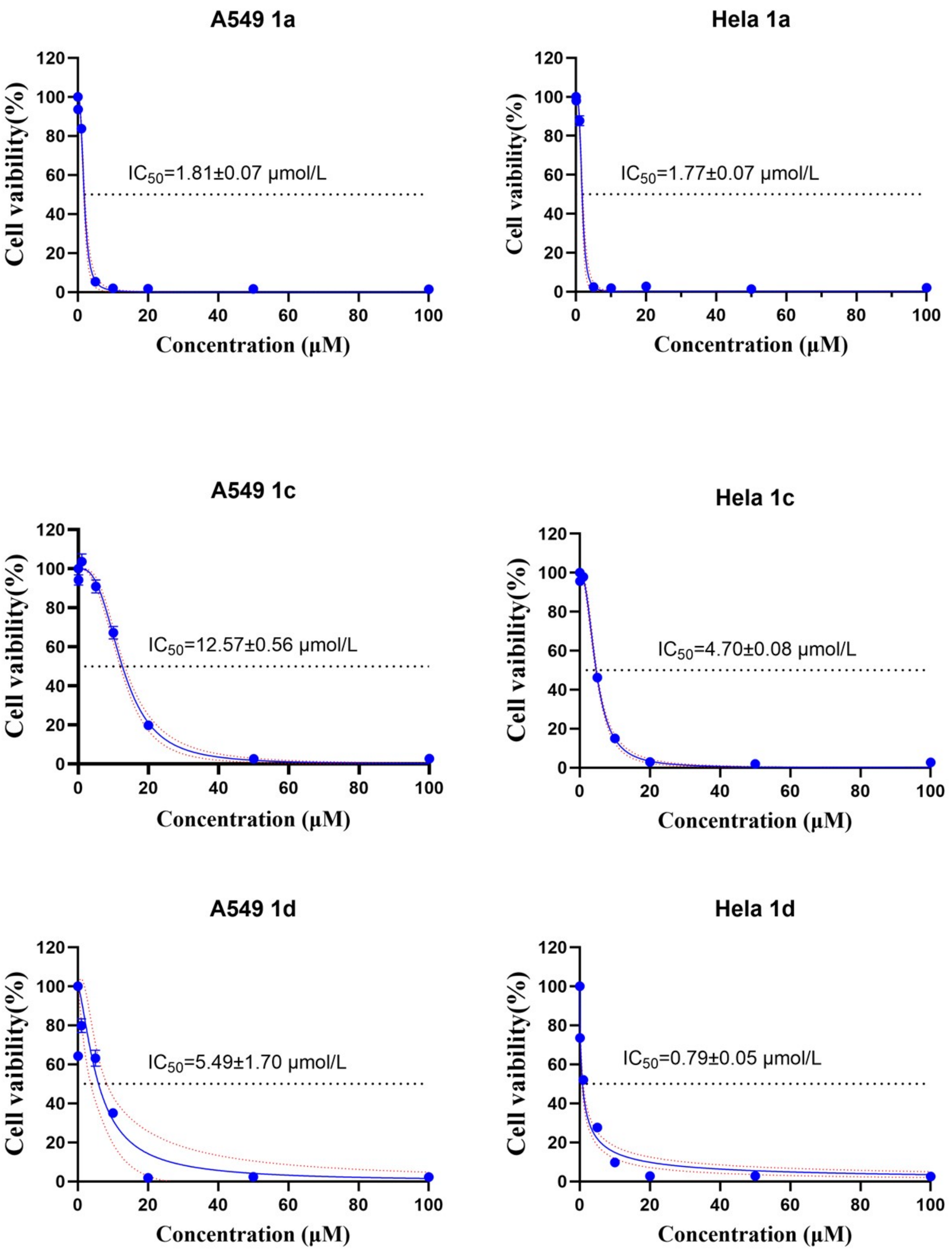


**Fig. S34** Antiproliferative activity of **1a**, **1c**, and **1d** against A549 and Hela (48 h, CCK-8 assays, $IC_{50}$, mean ± SD [μM]).

## 4. Inhibition of TrxR

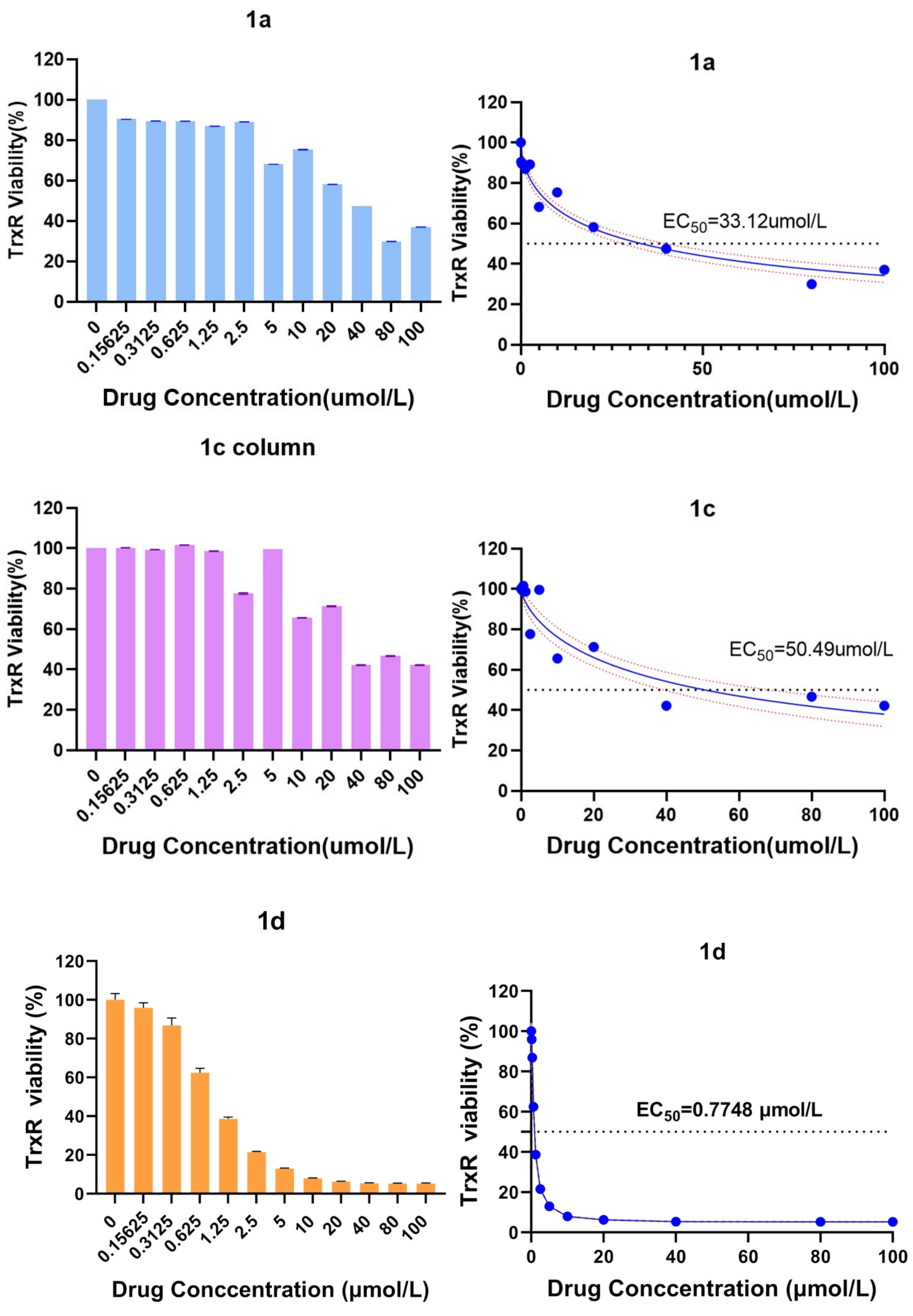


**Fig. S35** Inhibition to the activity of purified TrxR by complexes **1a**, **1c** and **1d**
